# Integrity of xylan backbone affects plant responses to drought

**DOI:** 10.3389/fpls.2024.1422701

**Published:** 2024-06-25

**Authors:** Félix R. Barbut, Emilie Cavel, Evgeniy N. Donev, Ioana Gaboreanu, János Urbancsok, Garima Pandey, Hervé Demailly, Dianyi Jiao, Zakiya Yassin, Marta Derba-Maceluch, Emma R. Master, Gerhard Scheepers, Laurent Gutierrez, Ewa J. Mellerowicz

**Affiliations:** ^1^ Umeå Plant Science Centre, Swedish University of Agricultural Sciences, Department of Forest Genetics and Plant Physiology, Umeå, Sweden; ^2^ Centre de Ressources Régionales en Biologie Moléculaire (CRRBM), University of Picardie Jules Verne, Amiens, France; ^3^ RISE Research Institutes of Sweden, Built Environment Division, Stockholm, Sweden; ^4^ Department of Chemical Engineering and Applied Chemistry Department, University of Toronto, Toronto, ON, Canada

**Keywords:** glucuronoxylan, drought stress, *Arabidopsis*, *Populus*, secondary cell wall, hyperspectral imaging, high-throughput phenotyping, cell wall integrity

## Abstract

Drought is a major factor affecting crops, thus efforts are needed to increase plant resilience to this abiotic stress. The overlapping signaling pathways between drought and cell wall integrity maintenance responses create a possibility of increasing drought resistance by modifying cell walls. Here, using herbaceous and woody plant model species, *Arabidopsis* and hybrid aspen, respectively, we investigated how the integrity of xylan in secondary walls affects the responses of plants to drought stress. Plants, in which secondary wall xylan integrity was reduced by expressing fungal GH10 and GH11 xylanases or by affecting genes involved in xylan backbone biosynthesis, were subjected to controlled drought while their physiological responses were continuously monitored by RGB, fluorescence, and/or hyperspectral cameras. For *Arabidopsis*, this was supplemented with survival test after complete water withdrawal and analyses of stomatal function and stem conductivity. All *Arabidopsis* xylan-impaired lines showed better survival upon complete watering withdrawal, increased stomatal density and delayed growth inhibition by moderate drought, indicating increased resilience to moderate drought associated with modified xylan integrity. Subtle differences were recorded between xylan biosynthesis mutants (*irx9*, *irx10* and *irx14*) and xylanase-expressing lines. *irx14* was the most drought resistant genotype, and the only genotype with increased lignin content and unaltered xylem conductivity despite its *irx* phenotype. Rosette growth was more affected by drought in GH11- than in GH10-expressing plants. In aspen, mild downregulation of *GT43B* and *C* genes did not affect drought responses and the transgenic plants grew better than the wild-type in drought and well-watered conditions. Both GH10 and GH11 xylanases strongly inhibited stem elongation and root growth in well-watered conditions but growth was less inhibited by drought in GH11-expressing plants than in wild-type. Overall, plants with xylan integrity impairment in secondary walls were less affected than wild-type by moderately reduced water availability but their responses also varied among genotypes and species. Thus, modifying the secondary cell wall integrity can be considered as a potential strategy for developing crops better suited to withstand water scarcity, but more research is needed to address the underlying molecular causes of this variability.

## Introduction

As a major adverse factor, drought impacts crops and forests, endangering yield, and causing important economic losses (D’Odorico et al., 2021; Dietz et al., 2021). With rising pressure on food and energy production entangled with water scarcity and increasing global drought events, tackling plants’ intricate and multifaceted physiological mechanisms toward better water management appears to be crucial. Drought stress alters whole plant physiology triggering adaptive changes in cell homeostasis and plant morphology that are qualified as drought avoidance, escape, acclimation, tolerance, and resistance (Gilbert and Medina, 2016). Growth of all organs is affected, ranging from the roots to increase water uptake, to the leaves to limit water loss, and to the whole vascular system to avoid cavitation (Brodribb et al., 2010; Gupta et al., 2020). Drought induces ABA signaling, resulting in stomatal closure to reduce evaporation which affects photosynthesis and reduces shoot growth, in osmolyte accumulation to counter cell’s dehydration stress and in root system increased growth to explore deeper soil. At the cellular level, drought-induced plasmolysis is sensed by mechanosensitive ion channels which regulate intracellular Ca^2+^ concentration triggering calcium signaling (Scharwies and Dinneny, 2019). Calcium signaling activates membrane NADPH oxidases RESPIRATORY BURST OXIDASE HOMOLOGUE (RBOH) D and F, generating apoplastic reactive oxygen species (ROS) which activate H_2_O_2_ signaling (Ravi et al., 2023). RBOH activation is additionally promoted by ABA through a series of phosphorylation relay events involving an array of protein kinases (Chen et al., 2021). The central role is played by sucrose nonfermenting1 (SNF1)-related protein kinase 2 (SnRK2) which is part of ABA receptor complex. Its activation by ABA triggers mitogen-activated protein kinase (MAPK) cascades, calcium-dependent protein kinases (CDPKs/CPKs), CALCINEURIN B-LIKE (CBL)-INTERACTING PROTEIN KINASEs (CIPKs), different receptor-like kinases (RLKs) and plasma membrane proteins. Among them, aquaporins, anion channels SLAC1 or QUAC1 and inward rectifying K^+^ channels regulate osmotic cell status and stomatal movement (Lim et al., 2015; Hsu et al., 2021). Peptide signaling is also involved in drought response. CLE25 peptide perceived by BARELY ANY MERISTEM1 and 3 (BAM1/3) triggers ABA biosynthesis (Takahashi et al., 2018), and RALF1 peptide perceived by FERONIA (FER) transduces signaling by ROP-GEF pathway (Chen et al., 2016). Moreover, the proteins with disordered domains that could acquire new functions in response to osmotic changes are thought to participate in drought stress perception (Cuevas-Velazquez and Dinneny, 2018; Scharwies and Dinneny, 2019).

Drought signaling pathways partially overlap with other signaling events including cell wall integrity (CWI) maintenance (Bacete and Hamann, 2020; Gigli-Bisceglia and Testerink, 2021; Swaminathan et al., 2022). Several malectin-domain receptor kinases, which are key transducers of CWI signaling, are induced by drought (Kumar et al., 2020; Wang et al., 2021; Li et al., 2022). Previous exposure to stress tends to increase resilience to different stresses upon subsequent exposures resulting in cross-stress tolerance *via* mechanisms jointly called priming or stress memory (Kerchev et al., 2020; Liu et al., 2022). Priming can involve epigenetic changes, accumulation of different protective or signaling compounds including hormones, or morphological adaptations to stress (Fleta-Soriano and Munné-Bosch, 2016; Harris and Mou, 2023). In agreement with these concepts, the modification of cell wall frequently results in increased stress resilience to biotic (Hernández-Blanco et al., 2007; Pogorelko et al., 2013; Pawar et al., 2016) and abiotic (Chen et al., 2005; Ramírez and Pauly, 2019; Reem et al., 2020) stresses and in activation of defense pathways (Caño-Delgado et al., 2003; Gallego-Giraldo et al., 2011; Reem et al., 2020). Increased drought tolerance in *lew2 Arabidopsis* mutants, impaired in *CesA8* encoding a member of the secondary wall cellulose synthase complex (Chen et al., 2005), and in lignin deficient plants (Huang et al., 2010; Gallego-Giraldo et al., 2011; Yan et al., 2018) suggest that secondary CWI maintenance could be involved in its mediation. Defects in xylan structure affecting the integrity of secondary cell walls of xylem leading to *irregular xylem* (*irx*) phenotype are also known to increase drought and/or freezing stress resistance. For example, xylan acetylation mutant *eskimo1/tbl29* and engineered plants with reduced xylan content and acetylation have increased ability to survive drought and freezing stress (Xin et al., 2007; Bouchabke-Coussa et al., 2008; Lugan et al., 2009; Ramírez et al., 2018; Yan et al., 2018). Similarly increased stress resistance was observed in xylan backbone and reducing end sequence biosynthetic mutants *irx14*, *irx9* and *parvus* (Keppler and Showalter, 2010; Ramírez and Pauly, 2019). The underlying mechanism remains elusive but seems to involve an indirect mechanism that require strigolactone biosynthesis (but not signaling) (Ramírez et al., 2018, 2019) or KAKTUS function (Bensussan et al., 2015), that mediate xylan integrity defects. Moreover, there is evidence that xylobiose which could result from xylan damage can act as a damage-associated molecular pattern (DAMP) (Dewangan et al., 2023). These observations indicate that xylan structure impairments could be perceived *via* CWI or DAMP signaling resulting in increased drought resilience. However, it is still unclear if different xylan defects in secondary cell walls cause similar drought responses, which would be expected if the same secondary CWI pathways were activated in these plants. Moreover, it is unknown if increased drought resilience is caused by the dwarf phenotype of the studied mutants which decreases water consumption. To answer these questions, we monitored growth and physiological responses to controlled drought exposure in transgenic plants with xylan backbone defects induced by various ways but specifically in secondary walls, to study: i) if the impairment of the xylan network in the secondary cell walls leads to physiological changes that mitigate the drought impact; ii) if the drought responses of different transgenic lines are comparable; and iii) if xylan-defect induced drought resistance is similar between herbaceous and woody plants. For the last objective, two well-established model plant species, *Arabidopsis thaliana* (*Arabidopsis*) and *Populus tremula* L. x *tremuloides* Michx. (hybrid aspen), having similarly impaired xylan, were studied in controlled drought conditions. These species were studied in high-throughput phenotyping facilities that implement a plethora of advanced technologies such as RGB imaging, chlorophyll fluorescence and hyperspectral imaging to capture subtle physiological changes. For *Arabidopsis*, this approach was combined with analyses of stomatal function and xylem conductivity offering a more holistic view of the drought response of xylan-impaired plants.

## Materials and methods

### Generation of expression vectors for plant transformation

The *Aspergillus nidulans* cDNA clones *GH10* (*AN1818.2*; GenBank: ABF50851.1) and *GH11* (*ANIA_03613*; NCBI_GeneID:2873037, XP_661217.1) encoding GH10 and GH11 xylanases, respectively, originally described by Bauer et al. (2006) and obtained from the authors were used to create plant expression vectors. In the case of *GH10*, the sequence of native *A. nidulans* signal peptide was exchanged to the hybrid aspen (*Populus tremula* L. x *tremuloides* Michx.) signal peptide from gene *PtxtCel9B3* (alias *PttCel9B*) (GenBank accession no. AY660968.1; Rudsander et al., 2003) with use of the following primers: OC9Bf1, OC9Br5, FC5f1, FC5r and FC5r1s (**Supplementary Table S1**) and the method described previously (Gandla et al., 2015). *GH11* was cloned with its native signal peptide using the primers FC4f1, FC4r1s, FC4r1 (**Supplementary Table S1**). The entry clones were created using the pENTR/D-TOPO cloning system (Thermo Fisher Scientific, Uppsala, Sweden), and the expression clones were obtained using the Gateway System (Thermo Fisher Scientific) in pK-pGT43B-GW7 (Ratke et al., 2015) for expression specifically in cells developing secondary cell walls driven by the wood-specific promoter (WP). The vectors (*WP::GH10* and *WP::GH11*) were introduced into competent *Agrobacterium tumefaciens* (Smith and Townsend, 1907) Conn 1942, strain GV3101 using electroporation.

### Intracellular localization of cloned fungal proteins in plant cells

Both xylanase constructs (with *PtxtCel9B3* signal peptide or with native *A. nidulans* signal peptide) were subcloned to binary destination vector pK7FWG2.0 (Karimi et al., 2002) using Gateway System (Life Technologies™) and primers listed in **Supplementary Table 1**. *Arabidopsis thaliana* Col-0 plants were transformed using the floral dip method (Clough and Bent, 1998). T2 seedlings were tested for the presence of GFP protein in plasmolyzed root tissue as described in Gandla et al. (2015). The confocal laser scanning microscope (Leica TCS SP2, Leica Microsystems, Wetzlar, Germany) used an excitation light at 488 nm produced by an argon laser and emission signals between 498 and 530 nm. To verify that the signal had the spectrum of GFP, the lambda scan was performed every 10 nm between 480 and 630 nm and compared to that of GFP (**Supplementary Figure S1**).

### *Arabidopsis* mutants used and generation of xylanase-expressing lines

Three T-DNA insertion lines SALK_057033 *(irx9–2*), SALK_046368 (*irx10*), and SALK_038212 (*irx14*), all in Col-0 background, were generously shared by the authors (Wu et al., 2009, 2010). The wild-type Col-0 was obtained from the Nottingham *Arabidopsis* Stock Centre (NASC).

*Arabidopsis* xylanase-expressing lines were obtained by *Agrobacterium*-mediated floral-dip transformation (Clough and Bent, 1998) of Col-0. Transformed seeds were grown on ½ Murashige & Skoog medium (½ MS) plates with kanamycin (50 μg ml^−1^) to select homozygous single insert lines by segregation. Two best-expressing lines were selected from six to seven independent lines of original WP::GH10 and WP::GH11 lines based on transgene expression levels determined by RT-qPCR.

### RT-qPCR analysis

RNA was extracted using TRI Reagent™ (Applied Biosystems, Bedford, MA, USA) from three 8 cm basal segments of mature stems each from an approx. 20 cm - tall plant. DNA was removed using (DNA-free™ DNA Removal Kit; Thermo Fisher Scientific, Uppsala, Sweden) and cDNA was synthesized with iScript™ cDNA Synthesis Kit (Bio-Rad Laboratories AB, Hercules, CA, USA). qPCR was carried out using 5 ng of cDNA per 10 μL reaction containing PCR primers at the 500 nM concentration (**Supplementary Table S1**) and 5 μL of SYBR green-labeled nucleotides in a LightCycler 480 SYBR Green I Master (Roche, Mannheim, Germany), and a 384-well plate reader (CFX384 Touch Real-Time PCR Detection System; Bio-Rad Laboratories AB). Expression levels were determined following the method described by Livak and Schmittgen (2001) using *ACTIN2* (*AT3G18780*) and *EF1α* (*AT5G60390*) as reference genes and presented relative to the line with the highest expression.

### Aspen lines used in the study

Hybrid aspen (*P. tremula* L. × *tremuloides* Michx., clone T89) was transformed by *A. tumefaciens* to obtain WP::GH10 and WP::GH11 lines as described in the accompanying paper (Sivan et al., 2024). Two or three lines with the highest expression were selected out of approx. 20 regenerated independent lines and propagated *in vitro* as described previously (Urbancsok et al., 2023).

### Growth conditions

#### Standard growth conditions for *Arabidopsis*

*Arabidopsis* were grown under long-day conditions (16/8 h light/dark; 100 μmol m^−2^ s^−1^ light intensity) with a day temperature of ~22°C and a night temperature of ~18°C. Pots were covered with plastic masks and the soil was mixed with vermiculite at 3:1 ratio (v:v).

#### Water withholding experiment

For the complete water withholding experiment, plants were grown in a standard growth conditions except that a mixture of soil, perlite, and vermiculite (2:1:1; v:v:v) was used (Yao et al., 2018). The soil mixture was saturated with water, left to drain for two days at which stage the soil was considered to contain water at field capacity, and then used for planting the cold-vernalized seeds. The plants were grown without watering for 40 days, and then they were watered for estimation of survival rate one week later. Plants were considered as surviving when they generated new shoots or leaves. The soil water content was assessed weekly by weighing individual pots and comparing their weight to the weight of these pots after complete drying of soil (the weight of plants was negligible). The soil water content was then related to the water content at field capacity.

#### Growth conditions in *Arabidopsis* high throughput phenotyping facility

**Figure 1:**
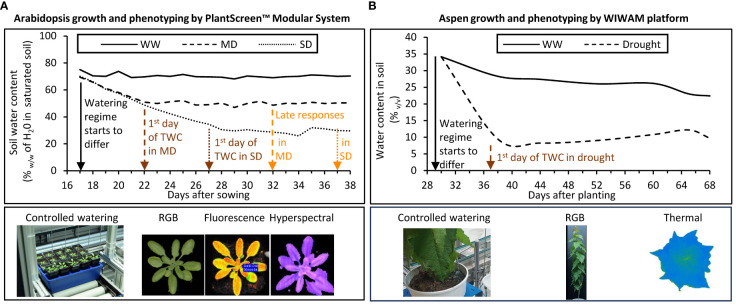
Watering regimes and image-based phenotyping instruments used to study *Arabidopsis* **(A)** and aspen **(B)** under controlled drought treatment. Different watering regimes were monitored through the maintenance of specific water content in soil by automated watering and weighing units on both platforms. Arrows indicate important dates to define the watering regimes: black arrow – date of change of watering regime; brown arrows – dates of Target Water Content (TCW) in soil corresponding to dates when early drought responses were recorded; orange arrows – dates when late drought responses were recorded. Red-Green-Blue (RGB) camera, chlorophyll fluorescence, hyperspectral (VNIR and SWIR), and thermal imaging units were used to detect changes in plant morphometric parameters and colors, photosynthetic activity, reflectance indices, and surface temperature, respectively. WW, well-watered; MD, moderate drought; SD, severe drought; VNIR, Visible and Near-Infrared; SWIR, Short-Wave Infrared.

The seeds were vernalized and sown into 250 ml pots filled with 110 g (+/- 5%) soil (NF U 44–551, Botanic, France) saturated with water to field capacity, and placed on Plantscreen™ (PSI, Photon Systems Instruments, spol. s r. o., Czech Republic) transportation trays for 20 pots. Each tray contained one plant per genotype. The genotypes were arranged in five different ways to account for a positional effect, each arrangement replicated 8 times. Plants were grown in Plantscreen™ Modular Facility for 38 days. Day/night conditions of 16 h/8 h with 140 μmol m^−2^ s^−1^ of light (Neptune LED, France) during daytime, temperatures of 19°C/15°C and an average relative humidity of 60%. The soil moisture content at field capacity was determined prior to the experimentation, and three different watering regimes (**Figure 1A**) were applied by daily adjusting watering. The control, i.e. well-watered (WW) conditions correspond 70% (w:w) of soil water content, the moderate drought (MD) and severe drought (SD) conditions correspond to 50% and 30% (w:w) of soil water content, respectively.

#### Growth conditions in aspen high throughput phenotyping facility

Aspen phenotyping platform (WIWAM Conveyor, custom designed by SMO, Eeklo, Belgium) was used described by Wang et al. (2022b). Plants were grown for nine weeks under 18 h/6 h (day/night) regime with 160–230 µmol m^-2^ s ^-1^ light intensity during the day, 22°C/18°C temperature, and the average air relative humidity of 60%. White light (FL300 LED Sunlight v1.1) and far-red light (FL100 LED custom-made, 725–735 nm) lamps from Senmatic A/S (Søndersø, Denmark) were used for illumination. *In vitro* propagated aspen saplings were planted in soil (K-jord, Hasselfors Garden AB, Örebro, Sweden) in 7 L plastic pots, watered to average soil moisture content of 25% to 30% (v:v), and covered immediately with transparent 8 L plastic bags for two weeks. Dry soil weight in each pot (determined using approximately 80 g of soil that was dried at 60°C for 6 days) was subsequently used by the WIWAM system to bring the humidity of soil to a configured target humidity (TH) level. After one week, the corners of the bags were cut open and after two weeks the bags were removed, and plants were watered automatically based on weight. After four weeks of growth, the soil humidity was reduced gradually over nine days by lowering the TH of WIWAM system from the starting 1.6–1.7 to 0.7 that corresponds to approx. 10% (v/v) soil moisture content for the drought-treated (D) trees (**Figure 1B**), similar to conditions used by Yu, et al. (2021). At the same time the soil humidity for the well-watered (WW) trees gradually increased over two days to TH of 1.9, corresponding to soil moisture content of 30–35% (v/v), the field capacity corresponding to TH 2.1. The weight of used water was replaced but weight of trees was not compensated by the system. D and WW treatments continued for an additional 3.5 weeks. During weeks three and four, all trees received 50 mL of 0.57% (v/v) fertilizer (SW Bouyant Rika S, SW Horto AB, Hammenhög, Sweden) applied every second day after imaging. Starting from the 7^th^ week, trees received 60 ml and 40 ml of fertilizer every other day for WW and D sets, respectively. As a pest control, plants were treated with Nemasys solution containing nematodes (*Steinernema feltiae*) (Bionema AB, Umeå, Sweden) against flies on the third and sixth week of growth. The weight of these solutions contributed to the total pot weight and the amount of water given to plants was then automatically adjusted. Soil moisture in the pots was periodically measured approximately 7 hours after watering using WET-2 sensor (HH2 reader, Delta-T devices, Cambridge, England).

### Phenotyping of *Arabidopsis*

#### High throughput *Arabidopsis* phenotyping analysis

Red-Green-Blue (RGB) and hyperspectral imaging were performed at 12:00 am every day in the PlantScreen™ Modular System. RGB images were obtained with a top-down camera (GigE μEye UI-5580SE-C/M - 5 Megapixels QSXGA Camera with 1/2” CMOS Sensor) to assess the morphometric parameters related to the rosette growth. Quadratic equations were fitted into growth kinetics data using Python v. 3.7, then, growth rates were calculated based on these quadratic equations in Microsoft Excel. Nine clusters of hues (Awlia et al., 2016) were used for the color segmentation analysis. Hyperspectral image acquisition was performed in the range of visible and near-infrared spectrum (VNIR, 380 – 870 nm) and shortwave infrared spectrum (SWIR, 930 – 1670 nm) with two different cameras, displaying a spectral resolution of 0.8 nm and 2 nm, respectively. Chlorophyll fluorescence images were taken during night-time, at 04:00 am, when the plants were dark-adapted. The kinetic chlorophyll fluorescence imaging unit (1.4 Mega-pixel High quality CCD sensor, 35 × 35 cm) used a pulse amplitude modulated (PAM) technique with the F_v_/F_m_ protocol, consisting of a F_0_ duration of 2 s followed by a saturating pulse of 800 ms set at 20% of intensity (~1137 µmol m^-2^ s ^-1^) according to the previous study (Awlia et al., 2016) and as recommended by Photon Systems Instruments. This protocol allowed to measure the F_0_, F_m_ and F_v_ parameters and to calculate the F_v_/F_m_ ratio, also known as the maximum quantum yield (QY_max) of photosystem II (PSII). Image segmentation and automatic raw data processing were done using the PlantScreen™ Analyzer software (v3.3.10.7, PSI, Drásov, Czech Republic). Different parameters used in this study calculated based on the spectral values are listed in **Supplementary Table S2**.

#### Cell wall analyses

A pool of eight debarked mature hypocotyls from approx. 25 cm-tall *Arabidopsis* plants per each line were freeze-dried and finely ground using a bead mill (Retsch MM400, Haan, Germany). The alcohol insoluble residue (AIR) was prepared by washing the powder successively with 80% ethanol, 70% ethanol, 95% methanol, methanol-chloroform 1:1 (v:v) mixture, and acetone before vacuum-drying (modified from Gandla et al., 2015). Approximately 70 ± 10 μg of powder from four technical replicates per pool were loaded onto a pyrolyzer with an autosampler (PY-2020iD and AS-1020E; Frontier Lab, Koriyama, Japan), which was linked to a GC/MS (7890A/5975C; Agilent Technologies AB, Santa Clara, CA, USA). The resulting pyrolysate was then separated and analyzed following the method by Gerber et al. (2012). Methanolysis-Trimethyl Silyl (TMS) analysis was carried out using 500 ± 30 μg of AIR material with an internal standard (10 μg of inositol) and five to seven technical replicates per line, alongside nine monosaccharide standards, as described in Gandla et al. (2015). Silylated monosaccharides were separated using GC/MS (7890A/5975C; Agilent Technologies AB). Raw data MS files were converted to CDF format in Agilent Chemstation Data Analysis (v.E.02.00.493) and then processed in R software (v.3.0.2; R Foundation for Statistical Computing) to perform data pretreatment steps, with baseline correction, chromatogram alignment, time-window setup, multivariate curve resolution (MCR) processing, and peak identification. The identification of 4-*O*-methylglucuronic acid was done following Chong et al. (2013).

#### Hypocotyl histological analysis

Cross sections (100 μm – thick) of *Arabidopsis* hypocotyls of approx. 20 cm-tall plants were obtained using a vibratome (VT100S; Leica Biosystems, Nussloch, Germany) and were stained for three minutes with 1% (w/v) phloroglucinol dissolved in 18% (v/v) HCl, rinsed in water, and observed using a bright field mode of a DMi8 microscope (Leica Microsystems, Mannheim/Wetzlar, Germany) equipped with a camera (Leica DFC9000GT). The proportions between areas of xylem 2 to the total xylem (xylem 1 + xylem 2) (Chaffey et al., 2002) were calculated for four hypocotyls per genotype from the three radii evenly spaced around the circumference per section (with ImageJ) of xylem 1 and total xylem, and assuming their circular shape.

#### Leaf water usage, water loss, stomatal analyses, and xylem conductivity in the stem

To assess the water usage per rosette area, the rosette areas of 40-days-old *Arabidopsis* were determined for ten plants per genotype from the RGB images, processed with scripts written in the object-oriented programming language Python (Van Rossum and Drake, 2009) at PlantCV library (Gehan et al., 2017) available at https://plantcv.readthedocs.io/en/stable/analyze_size/. The total amount of water used by each plant during 40 day-cultivation was determined by pot weighing as the plant weight was negligeable.

The leaf water loss test was performed as described by Lee et al. (2021) using six plants per genotype. Entire rosettes were cut from 3-week-old plants two hours after dawn, promptly weighed, and placed in open Perti dishes on a bench in ambient temperature (22°C). After 3 h, they were reweighed and then dried at 60°C for 6 hours to determine the dry weight. The water loss from the leaves was calculated by subtracting the final weight from the initial weight, and the difference was divided by the initial water content.

Stomatal density and pore area were analyzed on a fully developed green leaf from 17 ± 1cm-tall plants using four plants per genotype. For stomatal visualization, a thin layer of transparent nail polish was applied to cover the abaxial leaf surface (Wu and Zhao, 2017), and the resulting imprint was analyzed by a Leica DMI8 microscope (Leica Microsystems). The imprint was divided into four sections having an area of 0.28 mm^2^ and all stomates in each section were counted to determine stomatal density. The stomatal pore area was measured as described by Gonzalez-Guzman et al. (2012) using leaves sampled two hours after dawn. Three leaf discs, 5 mm in diameter, were dissected from the collected leaf of each of four plants and exposed to light for 2 h at 22°C in a 10 mM 2-(N-morpholino)ethanesulfonic acid (MES)/KOH buffer, pH 6.2, supplemented with 50 mM KCl to induce full stomatal opening at the start (0 min). Then, two discs were subjected to 10 μM abscisic acid (ABA), one for 30-min and another for 60-min, to induce stomatal closure. After each treatment, discs were transferred to a microscope slide and imaged using a Leica DMI8 light microscope (Leica Microsystems). The experiment was performed using 45 stomata measured per plant per treatment. Images were analyzed using ImageJ.

Xylem conductivity assay was adopted from the protocol used for rice (Wang et al., 2022a). Stems from eight 17 ± 1 cm-tall plants per genotype were dissected in water to avoid introducing air bubbles, placed vertically in PCR tubes filled with 200 μL of 0.5 mM rhodamine B (Sigma, 79754) for a 1-hour dye uptake period, and then divided into four successive three-cm sections and the side-shoots, which were weighed, frozen, and ball-milled into powder. The frozen powders were suspended in water at the same w/v concentration for each sample and 100 μL of each extract was analyzed using FluoroMax Plus spectrofluorometer (Horiba, Piscataway, New Jersey, USA), with excitation at 553 nm and emission spectrum measurements at 627 nm. In parallel, 100 μm - thick cross sections were cut from the stems between sections 3 and 4 using a vibratome (VT100S; Leica Biosystems) and observed under a DMI8 fluorescence microscope (Leica Microsystems) using 550 nm excitation.

#### Clustering of genotypes according to drought responses in *Arabidopsis*

Eight different *Arabidopsis* genotypes were examined in WW, MD, and SD conditions with respect to 18 traits, with measurements taken on the day of reaching the target soil moisture level (“early response”) and ten days later (“late response”). To evaluate effects of drought in each genotype, the measurements taken under MD and SD conditions were divided by the average values in WW conditions for each genotype. The clustering analysis was performed using the Ward Linkage method (Ward, 1963; Awlia et al., 2016). Between 24 to 37 replicates per line, occasion, and treatment were used. The computations were performed in Python 3.7 using the code available at the github website (https://github.com/bdallard/xylan_backbone_effects).

### Growth-related parameters in aspen

Plant height was recorded by the WIWAM system every second day during the imaging treatment as described by (Wang et al., 2022b). In addition, the WIWAM system acquired thermal top images of individual trees with the thermal camera FLIR A655sc (FLIR System Inc., Wilsonville, OR, USA). Mean pseudo-temperatures were computed in the object-oriented programming language Python (Van Rossum and Drake, 2009) using the PlantCV library (Gehan et al., 2017). The method used is available at https://plantcv.readthedocs.io/en/stable/analyze_thermal. At the end of experiment, the final stem height and diameter at the stem base were measured manually using a measuring tape and a caliper, respectively. The above-ground biomass was determined by weighing freshly collected plant stems with the leaves. To evaluate leaf morphology, leaf number 20 from the top was collected and its image was digitized using a scanner. The leaf area was then computed using ImageJ software, following the methodology of Schneider et al. (2012). For the belowground biomass determination, the pots were allowed to dry for 20 days, and then roots were cleaned, dried for additional ten days and weighed. Wood anatomical and mechanical traits were analyzed using SilviScan for samples dissected from the stem base using methods described previously (Urbancsok et al., 2023).

## Results

### *Arabidopsis* lines expressing fungal xylanases exhibited reduced xylan content, altered lignin content and *irregular xylem* phenotype similar to xylan backbone mutants

**Figure 2:**
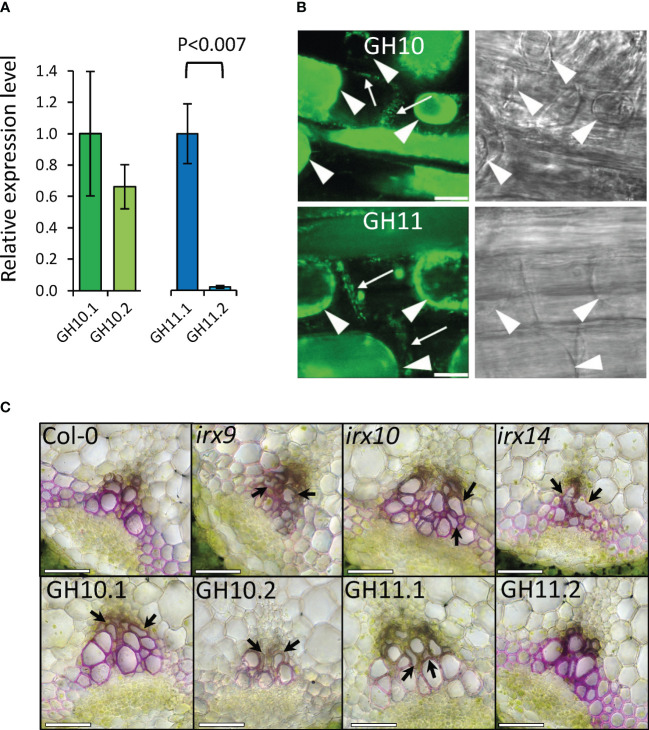
Expressing GH10 and GH11 xylanases in xylem cell walls of *Arabidopsis* induced *irregular xylem phenotype* similar to phenotype of *irx* mutants. **(A)** Transcripts of *Aspergillus nidulans GH10* (green bars) and *GH11* (blue bars) transgenes were detected in basal stem tissues of two independent lines expressing xylanases under control of wood-specific promoter (WP) (mean relative expression ± se; n=3; p-value according to t-test). **(B)** GH10:eGFP and GH11:eGFP recombinant proteins were detected in cell walls (arrows) in *Arabidopsis* root cells plasmolyzed with 20% mannitol. GFP channel (left) and the transmitted light channel (right). Plasmolyzed protoplasts are shown with arrowheads. Scale bar: 10 µm. **(C)** Stem vascular bundle cross-sections stained with rhodamine B showing *irregular xylem* phenotype (arrows). Scale bars = 50 μm.

To modify xylan in specifically in secondary walls, we expressed fungal GH10 and GH11 xylanases under control of aspen wood-specific promoter (WP) from aspen *GT43B* gene (Ratke et al., 2015). WP was shown to drive expression in cells depositing secondary cell walls in *Arabidopsis* (Pawar et al., 2016). The relative expression levels of transgenes in basal stem tissues assessed using RT-qPCR showed that the two selected lines of GH10 had similar expression levels, but for the GH11, the second most expressed line (GH11.2) had only 2.3% of the expression seen in the first line (GH11.1) (**Figure 2A**). The GH10 and GH11 xylanases were targeted to the apoplast by a signal peptide. To verify this targeting, 35S::GH10:eGFP and 35S::GH11:eGFP constructs were introduced to *Arabidopsis* and their root cells were plasmolyzed to reveal the GFP signal in cell walls (**Figure 2B**). Wild-type plants showed no fluorescence (**Supplementary Figure S1**). This shows that the signal peptides were capable of apoplastic targeting.

To confirm that the generated lines had altered xylan structure, we analyzed their matrix sugar composition in the secondary xylem of eight-week-old hypocotyls by acid methanolysis -TMS, and compared with that of *irx9*, *irx14*, and *irx10* mutants, with defects in xylan backbone elongation (Brown et al., 2007, 2009; Wu et al., 2009, 2010; Lee et al., 2010). The results (**Table 1**) showed that xylose content was lower compared to Col-0 for all transgenic lines and *irx* mutants but the line GH11.2, in agreement with its low level of relative *GH11* expression. In addition, the mannose content was increased in GH10 lines and *irx* mutants, and galactose and galacturonic acid increased in all lines but GH11.2 whereas glucuronic acid decreased in these lines which led to a higher ratio of methylated to unmethylated glucuronic acid, a defect previously reported in many xylan mutants (Brown et al., 2007, 2009; Lee et al., 2010; Wu et al., 2010). Thus, GH10 and GH11 expression affected xylan integrity similar to the defects seen in *irx9*, *irx10*, and *irx14* mutants, and led to compensatory changes in pectins and mannan. Pyrolysis-GC/MS (**Table 2**) analyses revealed a reduction in lignin content, in GH10 and GH1 lines, and in irx9 and irx10 mutants, compared to wild-type mainly driven by decrease in G-lignin. *irx14* mutant showed an opposite phenotype – increased total lignin content caused by an increase in S, G, and H lignin. *irx9* mutant was the only genotype with lower carbohydrates in addition to lower lignin content, which was partially compensated by increased S lignin and phenolics.

**Table 1:** Matrix sugar composition of cell walls in secondary xylem of eight-week-old hypocotyls of xylan-modified *Arabidopsis* based on acid methanolysis –TMS.

	Col-0	GH11.1	GH11.2	GH10.1	GH10.2	*irx9*	*irx10*	*irx14*
Arabinose	22.5±1.0	24.6±0.7	19.1±0.5	24.9±1.1	24.5±1.7	**32.2±2.4**	21.9±0.6	27.3±1.8
Rhamnose	14.3±0.2	**17.8±0.5**	13.2±0.2	15.4±0.5	16.5±1.0	**19.8±1.4**	16.0±0.5	**18.8±0.8**
Fucose	12.5±1.0	**16.9±0.9**	11.2±0.8	14.8±0.8	13.6±1.2	16.8±1.9	13.8±0.5	13.9±1.2
Xylose	189.8±5.3	**158.3±3.4**	193.4±6.0	**163.8±4.2**	**167.7±3.7**	**81.1±5.5**	121.6±2.8	**100.9±4.6**
Mannose	7.3±0.3	8.3±0.3	6.8±0.3	**10.0±0.5**	**10.2±0.7**	**11.3±1.0**	8.9±0.2	**19.0±1.2**
MeGlcA	1.7±0.1	1.7±0.1	1.8±0.1	2.0±0.1	2.1±0.1	1.5±0.1	1.6±0.0	1.6±0.1
Galactose	20.4±0.8	24.3±1.0	16.9±1.1	25.1±1.5	23.3±1.4	**29.5±2.5**	19.6±0.8	26.3±1.7
Galacturonic acid	66.0±2.2	**100.0±7.9**	61.3±3.9	**87.8±5.8**	83.2±4.7	**88.6±6.4**	76.8±2.2	84.1±6.0
Glucose	40.2±1.1	**60.0±1.9**	38.3±1.2	**46.2±1.9**	34.9±1.7	**19.1**±1.1	**53.5±1.7**	35.7±2.2
Glucuronic acid	4.9±0.2	**3.2±0.2**	5.2±0.3	**3.3±0.2**	**3.5±0.3**	**1.8±0.2**	**1.9±0.1**	**1.7±0.1**
Total	382±10	410±16	365±8	390±10	380±13	**302±22**	**330±8**	345±3
MeGlcA/GlcA	0.4±0.0	**0.6**±0.0	0.4±0.0	**0.6±0.0**	**0.6±0.0**	**0.8±0.0**	**0.9±0.0**	**0.9±0.0**

Data show sugar content (μg mg^-1^); means ± SE; bold font indicates means significantly different from Col-0 (Dunnett-test at P≤0.05). MeGlcA: 4-O-methylglucuronic acid.

**Table 2:** Cell wall composition in secondary xylem of eight-week-old hypocotyls of xylan-modified *Arabidopsis* analyzed by pyrolysis – GC/MS.

	Col-0	GH11.1	GH11.2	GH10.1	GH10.2	*irx9*	*irx10*	*irx14*
S lignin	2.6±0.1	2.7±0.3	2.6±0.1	2.3±0.0	2.6±0.1	**4.2±0.2**	3.0±0.4	**3.4±0.0**
G lignin	16.0±0.5	**11.1±0.3**	14.6±0.1	**14.0±0.1**	**13.1±0.1**	**11.5±0.5**	**9.4±0.7**	**17.6±0.6**
H lignin	0.9±0.0	1.00±0.1	0.8±0.1	0.9±0.0	**1.1±0.0**	1.0±0.1	1.1±0.1	**1.3±0.1**
Phenolics	0.5±0.0	0.8±0.1	0.5±0.0	0.5±0.0	0.7±0.0	**1.7±0.2**	**1.3±0.5**	0.6±0.0
Total lignin	20.0±0.5	**15.5±0.2**	**18.5±0.2**	**17.8±0.2**	**17.4**±0.2	**18.4±0.2**	**14.8±0.3**	**23.0±0.5**
Carbohydrates	75.0±0.6	75.7±1.7	75.9±0.6	76.7±0.2	75.7±0.6	**66.0±1.1**	75.3±3.6	72.4±0.4
Ratio S/G lignin	0.2±0.0	0.2±0.0	0.2±0.0	0.2±0.0	0.2±0.0	**0.4±0.0**	**0.3±0.1**	0.2±0.0
Carbohydrate/lignin	3.8±0.1	**4.9±0.2**	4.1±0.1	**4.3±0.1**	**4.4±0.1**	3.6±0.1	**5.1±0.3**	**3.2±0.1**

Data are means ± SE (%); bold font indicates means significantly different from Col-0 (Dunnett-test at P≤0.05).

The cell wall chemical composition analyses were carried out in the secondary xylem of hypocotyl, which contains two types of tissue, xylem 1 having lignified vessel elements and non-lignified parenchyma cells, and xylem 2 having lignified vessel elements and lignified fibers (Chaffey et al., 2002). To attest that the differences among the genotypes in cell wall composition are not driven by secondary xylem tissue composition, the anatomy of hypocotyls was analyzed and the areas of xylem 1 and xylem 2 were determined (**Supplementary Figures S2A, B**). We observed a similar ratio of xylem 2 to the total secondary xylem for all analyzed lines, except *irx9* which had increased content of xylem 2. This indicates that differences in chemical composition observed among the genotypes except *irx9* (**Tables 1**, **2**) reflect changes in xylem cell wall composition.

**Figure 3:**
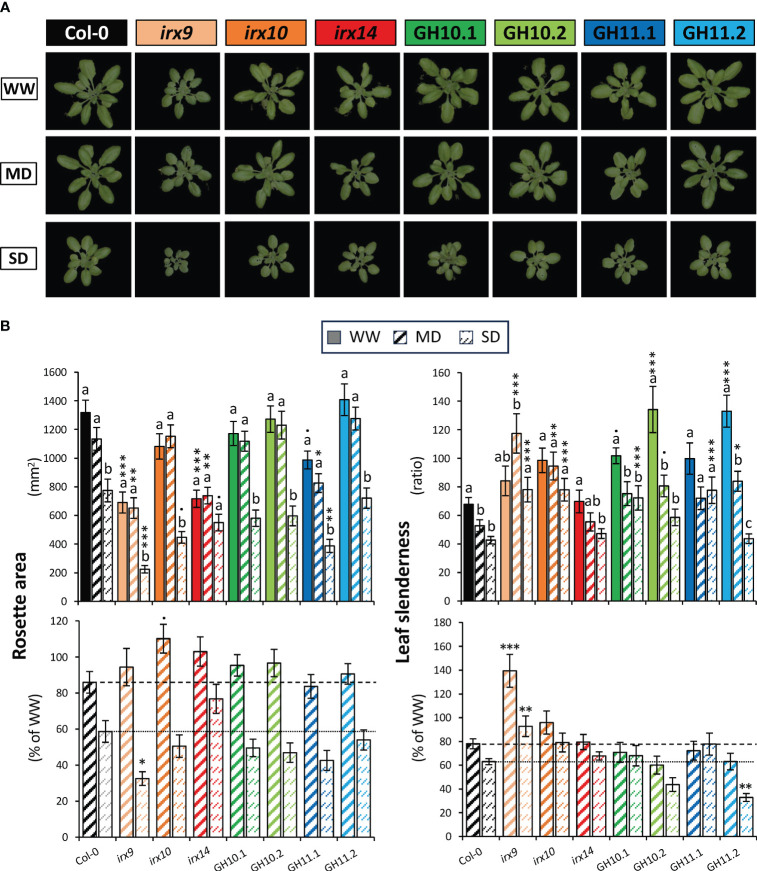
Effect of drought on rosette morphometric parameters of xylan-modified *Arabidopsis* lines at final stage of plant monitoring (38 days after sowing). **(A)** Representative images of rosettes under well-watered (WW), moderate drought (MD), and severe drought (SD) conditions **(B)** Bar graphs representing rosette area and leaf slenderness ratio in the different conditions of watering (top) and in MD or SD relative to the mean of each genotype in WW conditions (bottom). Means ± se; n: 24–37; • - P ≤ 0.1; * - P ≤ 0.05; ** - P ≤ 0.01; *** - P ≤ 0.001 for comparisons with Col-0 by Dunnett’s test; different letters indicate significant effects of treatments within the same genotype (Tukey’s test, P ≤ 0.05).

Next, to test if xylan deficiency in transgenic lines induced irregular xylem (*irx*) phenotype as known for *irx* mutants (Brown et al., 2007, 2009; Wu et al., 2009, 2010), we examined xylem morphology in the stem vascular bundles (**Figure 2C**). All lines but the low-expressed GH11.2 had the *irx* phenotype, like *irx* mutants. Majority of xylan-deficient lines grew like Col-0, only *irx9* and *irx14* showed approx. 50% reduction in rosette area, and GH11.1 line rosette area was slightly reduced (**Figures 3A, B** **;** data for WW conditions). On the other hand, a slight increase in leaf slenderness was observed in GH10.2 and GH11.2 lines, which could be interpreted as effect of minor xylan alterations.

### Xylan integrity status affected different aspects of plant reaction to drought stress in *Arabidopsis*

#### Controlled drought stress

To investigate responses of xylan-compromised plants to reduced soil water content, we reduced watering to reach approx. 50% and 30% of the water content at field capacity to simulate MD and SD, respectively. These levels were reached after 5 and 10 days, respectively, from the start of the drought treatment (**Figure 1A**). The soil water content level in the control WW condition was maintained at 70% of water content at field capacity. The plants were monitored until day 38 at which point the rosettes were filling the pots, but bolting was not initiated.

#### Rosette growth was less affected in xylan-modified *Arabidopsis* lines than in wild-type by moderate but more affected by severe controlled drought

Representative images of rosettes of plants grown for 38 days in WW, MD, and SD conditions are shown in **Figure 3A**. Drought did not induce any strong stress phenotypes as wilting, yellowing, or curling in any of the studied lines but it affected the morphometric parameters (**Figure 3B**). In the wild-type (Col-0), the SD treatment reduced rosette area by approx. 40% whereas only a slight not statistically significant area reduction was recorded in MD. *A contrario*, the *irx14* rosette area was not affected by MD or SD, revealing its resilience to the controlled drought. The *irx9, irx14* and GH11.1 rosette was smaller than that of Col-0 for all conditions, however, the reduction of rosette area by MD (but not SD) expressed as % of WW area tended to be less in all *irx* mutants and GH10 lines than observed for Col-0 (P_Contrast_ *_irx_*_and GH10_ *_vs_*_Col-0_<0.08). The leaf slenderness decreased in Col-0 when exposed to drought already in MD and no further change was observed in SD. In contrast, *irx9, irx10*, and GH11.1 did not exhibit reduced slenderness even under SD, but generally all xylan-impaired genotypes but *irx14* tended to have more slender leaves than Col-0 in all conditions. *irx9* reaction to drought was clearly different compared to Col-0 and other lines since it increased slenderness in MD conditions. In contrast, line GH11.2 showed a more pronounced reduction in slenderness in response to SD compared to Col-0.

**Figure 4:**
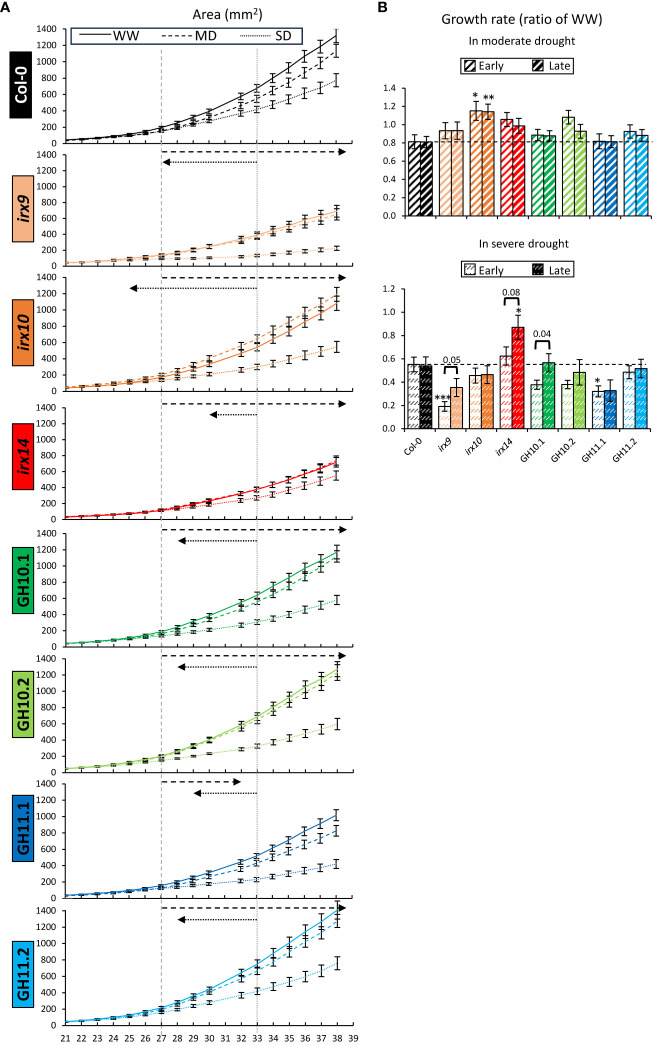
Kinetics of rosette growth in well-watered (WW), moderate drought (MD), and severe drought (SD) conditions revealed altered reactions to drought in xylan-modified *Arabidopsis* lines compared to Col-0. **(A)** Kinetics of rosette area from the 21^st^ to the 38^th^ day after sowing. Vertical gray lines indicate the days when MD-subjected Col-0 became smaller than in WW conditions (dashed line) and when SD-subjected Col-0 became smaller from MD-subjected Col-0 (dotted line) (P_t-test_ ≤0.05). Dashed and dotted arrows show how these dates differed for other genotypes. **(B)** Growth rate of the rosette in MD (top) or SD (bottom) relative to rate in WW conditions at early (on the first day of reached target water content) and late (ten days later) stages of drought treatment (see **Figure 1** for the dates). The growth rate was calculated as the first derivative at indicated days of the fitted trend lines (**Supplementary Table S3**). Data are means ± se; n: 24–37; * - P ≤ 0.05; ** - P ≤ 0.01; *** - P ≤ 0.001 for comparisons with Col-0 by Dunnett’s test. Horizontal brackets and P values indicate significant treatment effects within the same genotype (t-test, P ≤ 0.1).

The kinetics of rosette area showed that in Col-0, MD treatment negatively impacted the area five days after target drought level was reached (P_t-test ≤_0.05) which is shown by the vertical dashed gray line (**Figure 4A**). However, from day 37, the rosette growth recovered, which could be the result of adaptation to the MD. The rosette area became smaller under SD compared to MD six days after the target SD moisture level was reached (vertical dotted gray line). Interestingly, in all xylan-affected genotypes, the first sign of rosette growth reduction under MD was detected much later, whereas under SD it was detected earlier than in Col-0, as shown by the dashed and dotted arrows, respectively. This indicates a better drought tolerance of xylan-affected genotypes compared to wild-type when coping with MD but worse when coping with SD.

Quadratic equations were fitted into the kinetics data for rosette areas with the coefficient of determination R^2^ > 0.99 for all the fits (**Supplementary Table S3**). The rates of rosette growth (calculated as first derivatives of kinetics data) at early and late stages of MD adaptation relative to WW conditions were increased in *irx10* while other genotypes did not show any difference compared to Col-0 (**Figure 4B**). However, in SD, *irx14* had a higher growth rate relative to WW condition during late response, whereas the growth rate of *irx9* and GH11.1 at the early stage was decreased compared to Col-0. Interestingly, in three genotypes, *irx9, irx14*, and GH10.1, the speed of rosette growth increased at the late compared to early SD response, showing the adaptation to stress in these genotypes.

#### Effects of drought on fluorescence and reflected light spectra were altered in xylan-modified *Arabidopsis* lines

**Figure 5:**
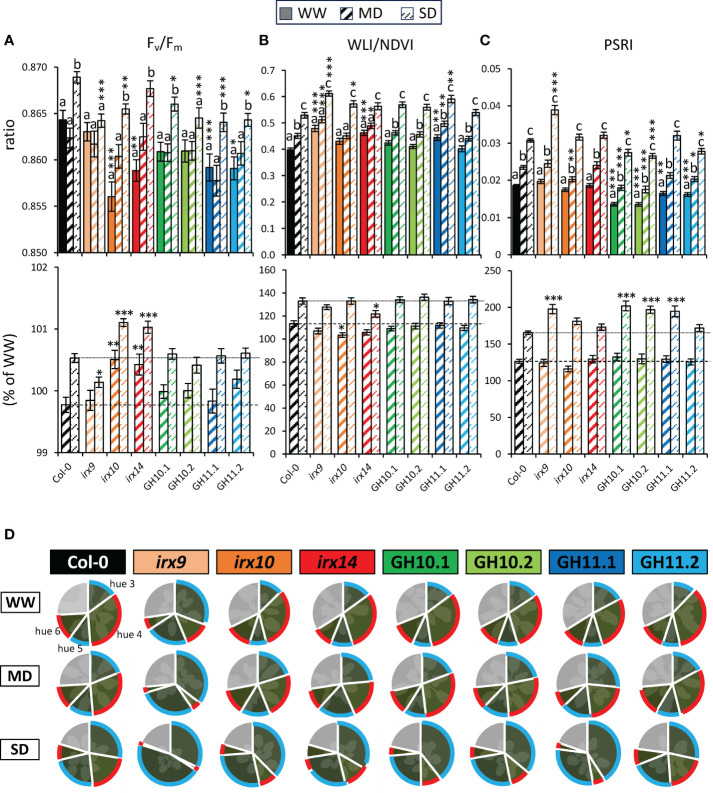
Parameters determined by fluorescence and reflected light spectra at the final stage of plant monitoring (38 days after sowing) in well-watered (WW), moderate drought (MD), and severe drought (SD) conditions revealed changes in reactions to drought of xylan-modified *Arabidopsis* lines compared to Col-0. F_v_/F_m_ ratios **(A)**, water loss index (WLI) divided by normalized difference vegetation index (NDVI) **(B)**, and plant senescence reflectance index (PSRI) **(C)** at different conditions (upper row) and in MD or SD relative to the mean of each genotype in WW conditions (lower row). **(D)** Percentage of four greenness hues from color segmentation analysis. In clockwise order: hue 3–6 (as shown for Col-0 WW) and hue 1 + 2 + 7 + 8+9 in grey (**Supplementary Figure S4**). Data in **(A–C)** are means ± se; n: 24–37; * - P ≤ 0.05; ** - P ≤ 0.01; *** - P ≤ 0.001 for comparisons with Col-0 by Dunnett’s test; different letters indicate significant treatment effect within the same line (Tukey’s test, P ≤ 0.05).

To study the subtle differences in plant physiology in drought stress conditions among the different xylan-modified plants, we analyzed their spectral values obtained using different cameras available in the PlantScreen™ system (**Figure 1**). The quantum yield of photosystem II, also referred to as the F_v_/F_m_ ratio, is the most commonly used parameter to assess photosynthesis efficiency from the fluorescence camera measurements and changes in this parameter occur under drought stress (Jansen et al., 2009; Yao et al., 2018). In Col-0, F_v_/F_m_ increased under SD compared to MD or WW conditions through the treatment (**Figure 5A**; **Supplementary Figure S3A**). Strikingly, F_v_/F_m_ was lower in all xylan-modified plants than in Col-0 in one or more of the analyzed growth conditions. Moreover, the *irx* mutants exhibited differences in changes in this parameter in response to drought as expressed in % to WW conditions, compared to Col-0. *irx9* plants showed a smaller increase in F_v_/F_m_ under SD conditions whereas *irx10* and *irx14* mutants exhibited a greater increase than Col-0 in both, MD and SD conditions. This highlights that xylan-modified *Arabidopsis* responds in opposite directions between *irx9* versus *irx10* and *irx14*. Overall, based on the F_v_/F_m_ parameter, all xylan-modified *Arabidopsis* lines showed differences in photosystem II efficiency compared to Col-0, with *irx10* and *irx14* having a stronger reaction to drought compared to Col-0, contrasting with *irx9*.

The water loss analyzed by the normalized water loss index (WLI/NDVI) increased significantly between WW, MD, and SD conditions for Col-0, whereas the reaction of *irx10* and *irx14* plants was reduced in MD or SD conditions, respectively (**Figure 5B**). This is in agreement with the previous conclusions that *irx10* and *irx14* might be tolerant to MD and SD, respectively. However, these mutants as well as *irx9* and GH11.1, but strikingly not GH10 lines, had increased WLI/NDVI index compared to Col-0 under at least one or more growth conditions.

The Photochemical Reflectance Index (PRI) that is linked to photosynthetic parameters (Gamon et al., 1992) was decreased during late stage of SD for all genotypes but *irx14* (**Supplementary Figure S3**). The Plant Senescence Reflectance Index (PSRI) which corresponds to the carotenoid-to-chlorophyll ratio and serves as an indicator of leaf senescence (Merzlyak et al., 1999) progressively increased under drought in all studied genotypes and was the highest in *irx9* under SD (**Figure 5C**). Lines expressing either GH10 or GH11 had lower PSRI compared to Col-0 in WW conditions and in one or more drought conditions. It was also true for *irx10* in MD conditions. In addition, whereas the amplitude of response to MD expressed in % of WW status was uniform across all the genotypes, the response to SD was much increased in *irx9*, both GH10 lines and GH11.1 compared to Col-0. To conclude, the xylanase-expressing lines exhibited reduced senescence in WW and MD but these lines and *irx9* showed a stronger induction of senescence under SD conditions.

Drought also impacted the leaf colors depicted through color segmentation analysis. There was a shift in greenness hues with the plant development, especially between the 26th and 30th day after sowing, which was more pronounced under SD conditions (**Supplementary Figure S4A**). At the end of the experiment in drought conditions, hue 3 and hue 5 became most pronounced whereas hues 4 and 6 became reduced, and therefore these changes could constitute the senescence markers. Hues 3 and 5 were intensified by the drought severity, whereas hues 4 and 6 showed an opposite trend (**Figure 5D**; **Supplementary Figures S4B, C**). All xylan-affected genotypes except *irx9* reacted to SD and in some cases also MD with increased magnitude compared to Col-0. *irx9* displayed a particular phenotype showing very high levels of hue 3 + 5 versus hue 4 + 6, and the strongest decrease in hue 4 + 6 in drought conditions. In general, the analysis of color segmentation highlighted *irx9* having the most distinct phenotype and most prominent changes under drought but overall, we observed a general pattern of a stronger reaction to drought for all xylan-affected lines compared to Col-0, especially under SD.

#### Ward linkage clustering of different phenotypic parameters and genotypes allowed complexity reduction

**Figure 6:**
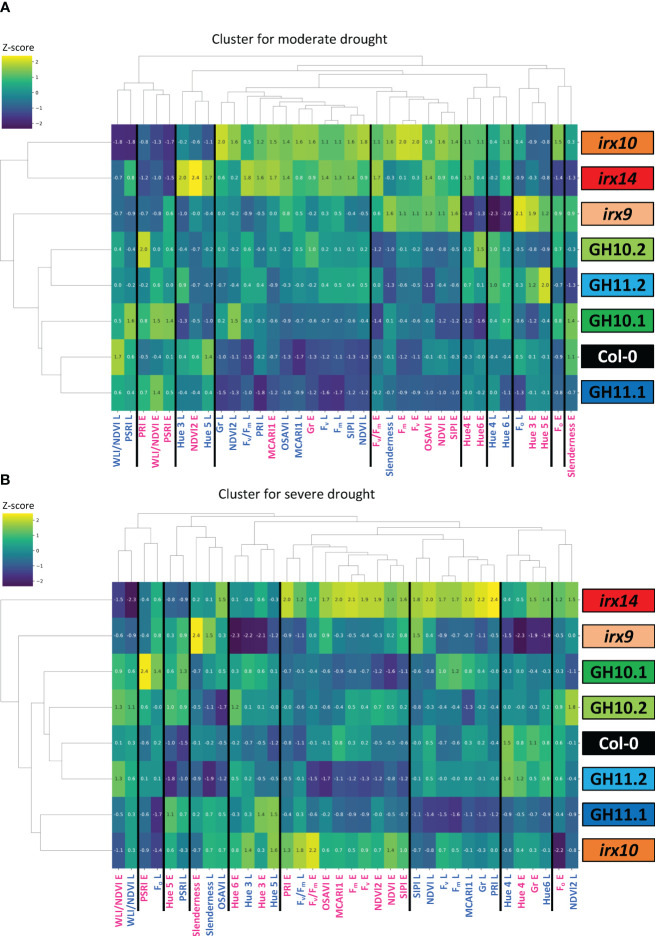
Clustering of genotypes according to their early and late responses in analyzed parameters to moderate **(A)** and severe **(B)** drought. Eighteen traits (**Supplementary Table S2**) measured on the first day when the target water content level was reached (E; early response) and ten days later (L; late response), expressed as ratios to their average values in well-watered conditions (**Supplementary Table S4**) were used for clustering with the Ward Linkage method. Values represent averages of 24–37 biological replicates per occasion, genotype, and treatment. Gr, Growth rate; MCARI, Modified Chlorophyll Absorption in Reflectance Index; NDVI, Normalized Difference Vegetation Index; OSAVI, Optimized Soil-Adjusted Vegetation Index; PRI, Photochemical Reflectance Index; PSRI, Plant Senescence Reflectance Index; F_v_/F_m_, maximum quantum yield of photosystem II; SIPI, Structure Insensitive Pigment Index; WLI, Water Loss index.

To evaluate overall similarities and differences in drought responses of different xylan-integrity compromised genotypes, we used the Ward linkage clustering method (Ward Jr., 1963; Awlia et al., 2016). Eighteen parameters (**Supplementary Table S2**) measured by PlantScreen™ system at early and late stages of exposure to MD and SD were expressed as ratios relative to WW conditions (**Supplementary Table S4**) and were used to construct two clusters, one for the MD and the other for the SD responses (**Figure 6**).

Some parameters, for example, hues 4 and 6, MCARI and the growth rate in MD, as well as the slenderness, the hues 3 and 4, the F_v_/F_m_, and WLI/NDVI in SD, clustered together at early and late stage of droughts, which indicates that these parameters were stable through the MD and SD treatments, respectively. These parameters provide potential predictive indicators for the plant behavior during stress. In contrast, some other parameters, such as NDVI and NDVI2, SIPI, OSAVI, and the fluorescence parameters F_o_, F_m_, and F_v_, showed clustering according to early and late drought responses, in both SD and MD, indicating temporal differences in plant responses to drought.

In MD, *irx10* and *irx14* mutants were grouped together, separately from the xylanase-expressing lines and Col-0 which formed a cluster, and the *irx9* mutant stood alone. In SD, the lower expressing line GH11.2 was clustered with Col-0 and GH10 lines, while the GH11.1 line clustered with *irx10* mutant. *irx9* and *irx14* were not clustered with any other lines and exhibited opposite parameter values. This regrouping shows that the reaction to drought by xylan integrity-compromised plants depended on the intensity of drought. Interestingly, GH10 and GH11 xylanases induced different responses in SD. To conclude, the physiological responses to drought as monitored by these various parameters differed in genotypes characterized by xylan integrity defects and depended on drought intensity and duration.

### Xylan integrity-compromised plants survived better the complete withholding of watering than wild-type

**Figure 7:**
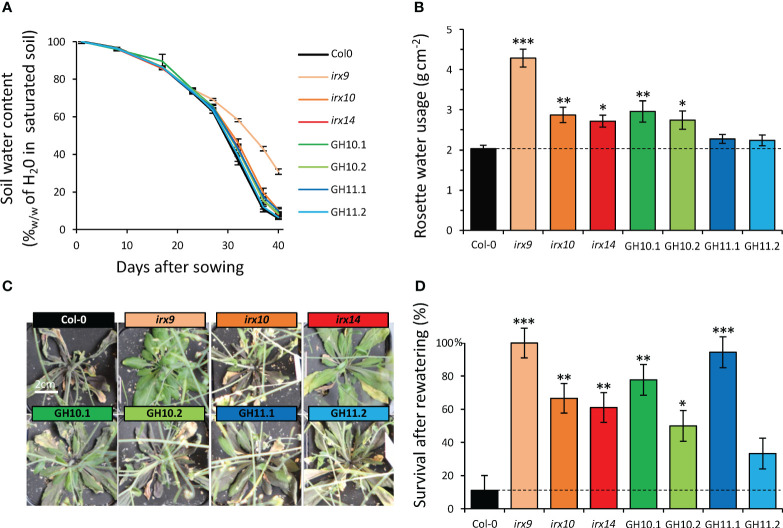
Better survival of xylan-modified *Arabidopsis* lines after complete withholding of watering and subsequent rewatering. **(A)** Changes in soil water content during water withholding treatment. **(B)** Cumulative water usage per rosette area during 27 days of growth without watering. **(C)** Representative images of rosettes grown without watering for 40 days. **(D)** Survival rate one week after rewatering following 40 days of drought. Data in **(A, B, D)** are means ± se; n= 10; * - P ≤ 0.05; ** - P ≤ 0.01; *** - P ≤ 0.001 for comparisons with Col-0 by Dunnett’s test in **(B)** or Fisher exact test in **(D)**.

To extend our understanding of the role of xylan in drought resilience, we subjected xylan-impaired plants to complete water-withholding for 40 days and subsequent re-watering – mimicking the real field situation. Although both *irx9* and *irx14* exhibited decreased rosette size compared to other genotypes (**Figures 3A, B**), thus were expected to use less water, the soil water depletion was slower only in pots with *irx9* compared to pots with other plants and this mutant was the only one that did not suffer any drought stress (**Figure 7A**). All *irx* mutants and GH10 lines used more water per rosette area compared to Col-0 with *irx9* showing the highest water usage (**Figure 7B**). After 40 days without watering, wild-type plants exhibited leaf yellowing and withering but these symptoms were mitigated in xylan-affected plants, especially in *irx9*, *irx14*, GH10.1, and GH11.1 (**Figure 7C**). After re-watering, all xylan-modified genotypes but GH11.2 showed a higher rate of survival compared to Col-0 (**Figure 7D**).

### Physiological parameters of xylan-modified *Arabidopsis* plants observed under well-watered conditions that could explain their reaction to drought

#### Xylan-modified plants exhibited reduced water evaporation and altered stomatal characteristics

**Figure 8:**
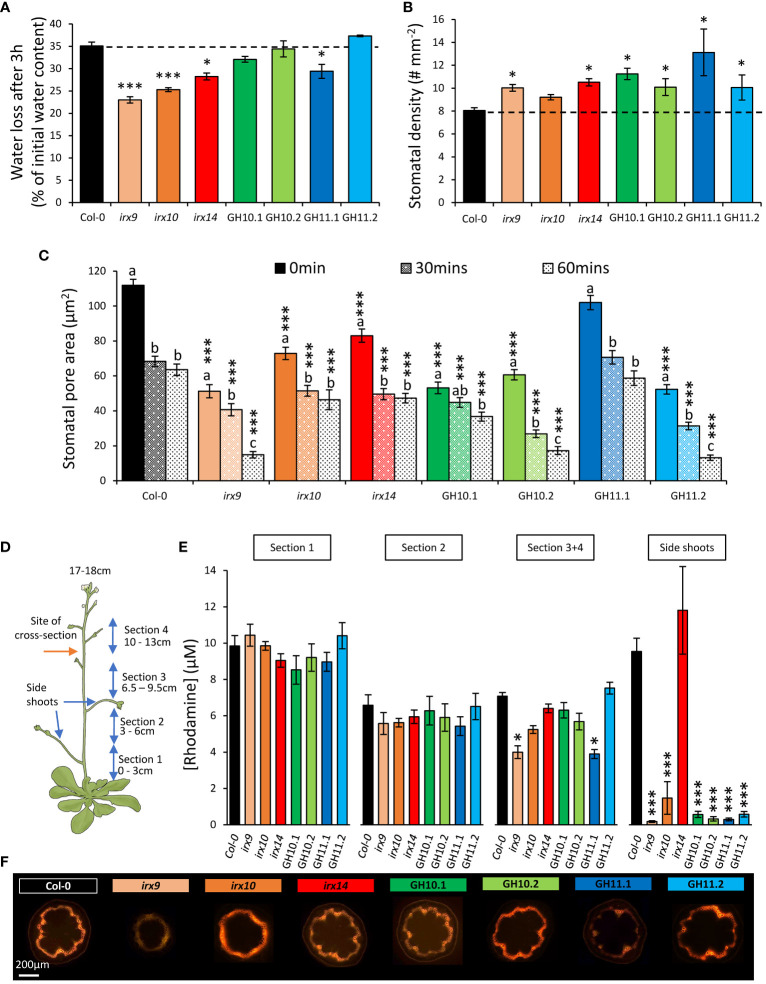
Xylan-modified *Arabidopsis* lines grown in well-watered conditions showed altered physiological parameters related to water evaporation and xylem conductivity. **(A)** Water loss from detached rosettes of 3-week-old plants. **(B)** Stomatal density. **(C)** Stomatal pore area measured in epidermal peels after 2 h light exposure in 50 mM KCl to induce full opening (0 min), followed by 30- and 60-min incubation in 10 μM abscisic acid (ABA) to induce closing. **(D, E)** Water conductivity in the main stem of 17 cm–tall plants determined by rhodamine uptake. Schematic representation of plants used for the conductivity analysis **(D)** and rhodamine released to 100 μL of water by 2 mg of tissue of successive stem sections and side-shoots after 1 h of uptake of 0.5 mM rhodamine **(E)**. **(F)** Representative images of stem cross sections made between sections 3 and 4, as shown with a red arrow in **(D)**, after 1 h of rhodamine uptake. Data in **(A–C, E)** are means ± se, n=10 for **(A)**, 4 for **(B)**, 4 x 45 (plants x stomates) for **(C)**, and 8 for **(E)**; * - P ≤ 0.05; *** - P ≤ 0.001 for comparisons with Col-0 by Dunnett’s test. Different letters in **(C)** indicate differences among different durations of ABA treatment within the same line (Tukey’s test, P ≤ 0.05).

Plants with a higher capacity to keep their water in their leaves might be more drought tolerant. To estimate how easily the plants lose water from detached leaves we exposed their leaves to ambient atmosphere for three hours. The percentage of lost water was reduced in *irx9*, *irx10*, and *irx14* mutants and in the line GH11.1 compared to Col-0 (**Figure 8A**). Leaf water loss depends on the leaf stomatal function (Juenger and Verslues, 2023). Interestingly, we observed that all xylan-modified plants but *irx10* showed a significantly higher density of stomata than Col-0 (**Figure 8B**) and the size of their pores, except for GH11.1, were reduced (**Figure 8C**). To investigate the stomatal capacity to close in reaction to ABA, we measured pore sizes after 30 min and 60 min exposure to ABA. We found that the lines with decreased pore size prior to ABA treatment maintained smaller pore sizes after exposure to ABA. Moreover, *irx9, irx10* and GH10.1 did not show a pronounced reaction to ABA after 30 min exposure. The closure was induced in *irx9* upon longer ABA exposure (60 min), but not in *irx10* or GH10.1. These measurements highlight the unique nature of each xylan-modified line while also revealing some similarity in terms of parameters determining stomatal transpiration. However, it is still unclear why some xylan-modified plants exhibit improved drought survival and reduced leaf water loss.

#### Vasculature conductivity was impaired in most xylan-modified *Arabidopsis* lines

We hypothesized that water loss and drought survival phenotypes could be related to changes in xylem conductivity, especially that most xylan-compromised lines described here exhibited *irregular xylem* phenotype (**Figure 2C**). Therefore, we measured xylem conductivity by monitoring rhodamine uptake into four successive stem sections and side shoots as shown in **Figure 8D**. Based on dye concentration and dye staining of the stem cross-sections, both *irx9* and GH11.1 conducted significantly less rhodamine into the upper stem part (sections 3 and 4) than Col-0, indicating a reduced capacity for stems to conduct water in these genotypes, whereas all lines but *irx14* had impaired transport to the side shoots (**Figures 8E, F** **).**

### Xylan integrity-impairment strongly affected aspen growth and its drought responses

To gain information on the impact of xylan modification on drought resilience in woody plant species, we investigated drought responses in aspen plants harboring the same transgenes (GH10 and GH11 xylanases under the control of WP) and plants affected in expression of GT43B and C clade genes (homologous to IRX9 and IRX14) involved in xylan backbone elongation (Ratke et al., 2018). The lines with different transgene expression levels are indicated by letters: from a – the strongest to c – the weakest. The phenotypes of xylanase-expressing lines are described in detail in the accompanying paper (Sivan et al., 2024).

**Figure 9:**
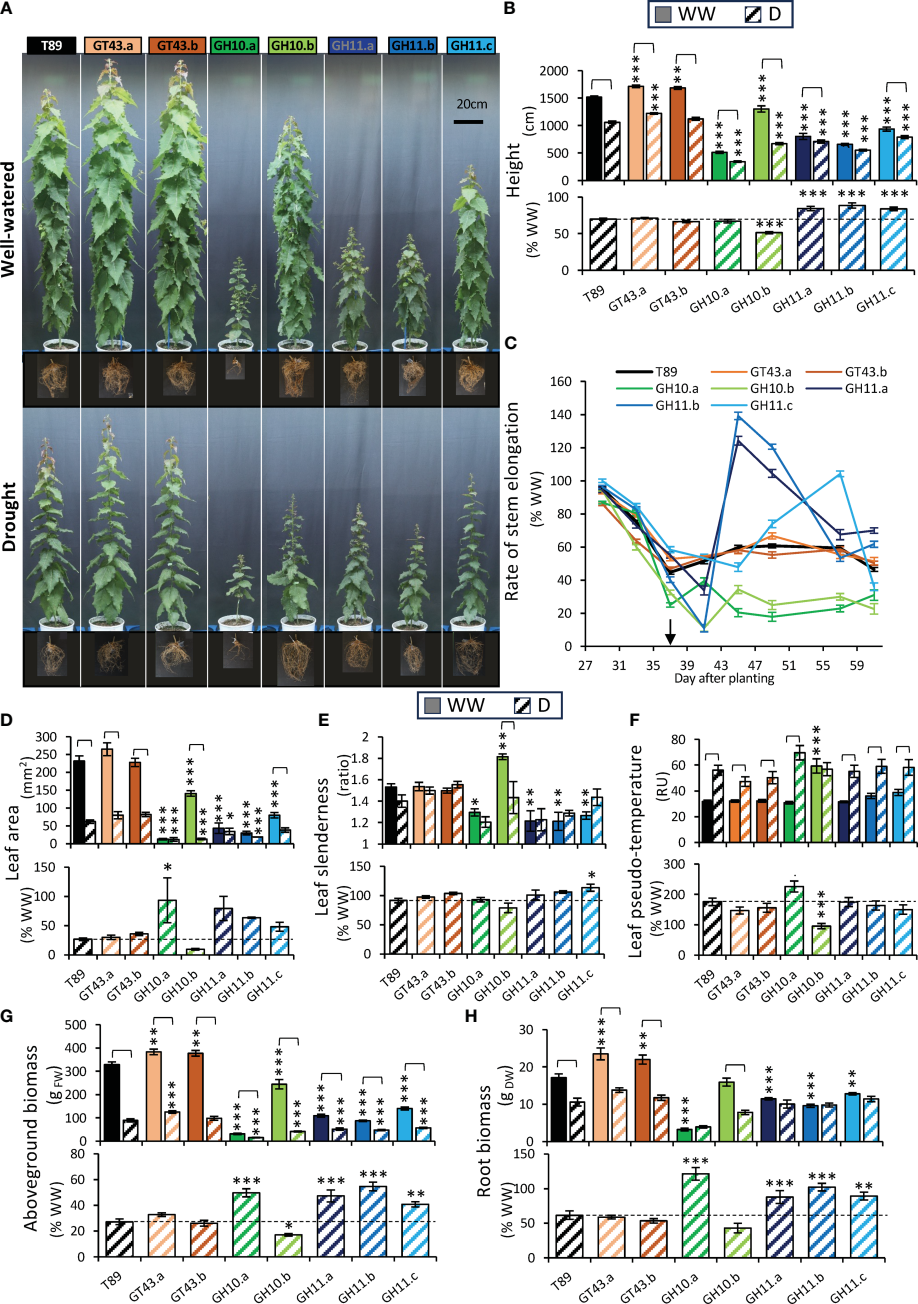
Effect of drought on growth of xylan-modified aspen lines. **(A)** Representative images of the trees and their roots under well-watered (WW) and drought (D) conditions at the end of drought treatment (65 days after planting). **(B)** Stem height 61 days after planting in WW and D conditions (upper row) and in D relative to mean height in WW conditions (lower row). **(C)** Rate of stem elongation of D-treated plants relative to average rate in WW condition revealed different responses to drought of GH10- and GH11-expressing lines. The arrow indicates the day when the target drought water content level was reached. **(D, E)** Morphology of the 20^th^ leaf from the top 65 days after planting. Leaf blade area **(D)** and slenderness ratio **(E)** in WW and D conditions (upper row) and in D relative to WW conditions (lower row). **(F)** Pseudo-temperature of the canopy from top IR camera 40 days after planting in WW and D conditions (upper row) and in D relative to WW conditions (lower row). Aboveground **(G)** and belowground **(H)** biomass 65 days after planting in WW and D conditions (upper row) and in D relative to WW conditions (lower row). Data in **(B–H)** are means ± se; n= 6–11; * - P ≤ 0.05; ** - P ≤ 0.01; *** - P ≤ 0.001 for comparisons with wild-type (T89) by Dunnett’s test; significant effects of drought are shown by brackets (t-test, P ≤ 0.05), RU, relative units.

Plants were subjected to a controlled drought regime (**Figure 1B**). At the end of the drought treatment, the morphology of wild-type plants (T89) was severely altered compared with plants in the WW conditions (**Figure 9A**) with all measured growth parameters (height, leaf size, above- and belowground biomass) reduced, whereas leaf slenderness was not affected (**Figures 9B, D, E, G, H**). The rate of stem elongation decreased upon drought treatment to approx. 50% of the rate in WW conditions (**Figure 9C**) and the water usage per plant was reduced to approx. 20% (**Supplementary Figure S5**), with concomitant increase in leaf temperature (**Figure 9F**).

GT43 lines were taller than T89 under WW conditions and had higher above- and belowground biomass (**Figures 9A, B, G, H**) in agreement with previous reports (Ratke et al., 2018). Better growth was maintained under drought conditions in line GT43.a. The reaction of GT43 lines to drought was generally similar to T89 for all analyzed growth parameters (**Figures 9A–H**). We concluded that in aspen with mildly reduced xylan content due to the wood-specific downregulation of GT43B and C genes leads to better-growing trees that cope with drought similarly as the T89.

The lines expressing GH10 and GH11 xylanases in the wood-forming tissues grew significantly worse than wild-type, showing reduced height, leaf size, and above- and belowground biomass in WW conditions (**Figures 9A, B, D, G, H**). The shoot growth of line GH10.b (but not line GH10.a which showed a very strong dwarf phenotype) was more affected by drought than that of T89 considering height, leaf size, and shoot biomass, whereas the shoot growth of GH11 lines was less affected. Noteworthy, the line GH10.a did not reach the target water stress during the experiment so its drought responses are not comparable with those of other lines. All three GH11 lines and GH10.a line showed almost no effect of drought on root biomass in contrast to T89 (**Figure 9H**). The rate of stem elongation under drought expressed in % of the rate in WW plants was more affected in GH10 lines than in T89, but less affected in GH11 even though this advantage appeared to be transient (**Figure 9C**). This was mirrored by the change in daily water usage per plant (**Supplementary Figure S5**). The leaves formed under drought stress in all xylanase-expressing lines but GH10.a were visibly smaller, as in wild-type (**Figure 9A**) but the measurements carried on leaf 20 (**Figure 9D**) did not reflect this phenotype in very dwarf plants because leaf 20 was formed under WW conditions in these plants. The leaves formed under drought were less slender than those formed in WW conditions in line GH10.b, whereas leaves of lines GH11 did not differ in slenderness between drought and WW conditions similarly as in T89 (**Figure 9E**). Despite the increased drought inhibition of shoot elongation in the GH10.b line compared to T89, its shoot temperature was not affected while it was almost doubled in all other lines (**Figure 9F**). This indicates a higher transpiration rate per leaf area in this line compared to other genotypes. Thus, the GH10 and GH11 lines differed in many aspects of their shoot and root growth responses to drought from those observed in T89.

**Figure 10:**
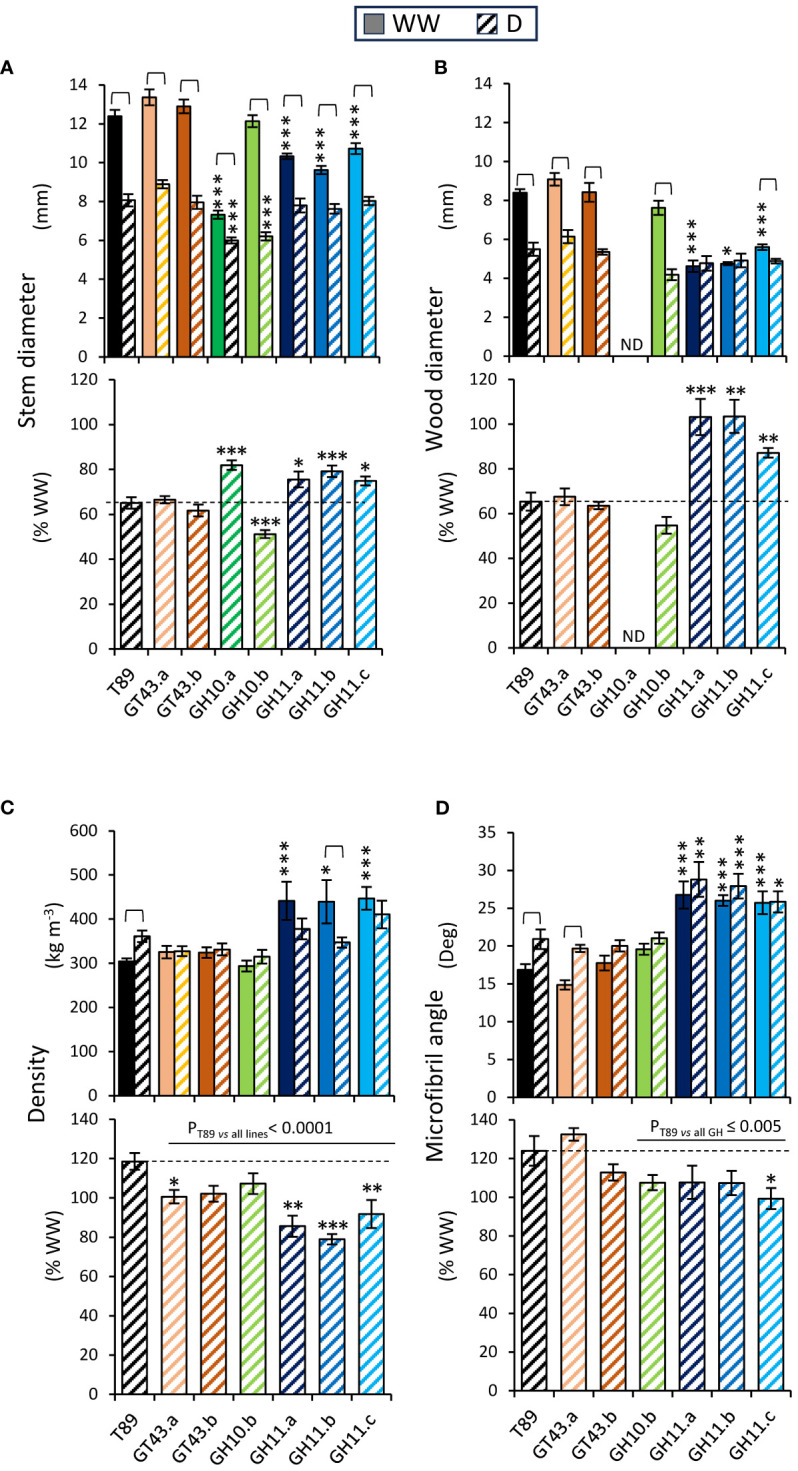
Analysis of stem diameters and wood properties measured at the stem base after 65 days of growth under drought (D) and well-watered (WW) conditions revealed different responses to drought of xylan-modified aspen lines compared to wild-type (T89). **(A)** Stem diameter measured with a caliper in WW and D conditions (upper graph) and in D relative to WW conditions (lower graph). **(B)** Diameter of wood core measured by SilviScan in WW and D conditions (upper graph) and in D relative to WW conditions (lower graph). **(C, D)** Wood properties determined by SilviScan analysis. Wood density in WW and D conditions (upper graph) and in D relative to WW conditions (lower graph) **(C)**, and microfibril angle in WW and D conditions (upper graph) and in D relative to WW conditions (lower graph) **(D)**. Data in **(A–D)** are means ± se; n= 6–11; * - P ≤ 0.05; ** - P ≤ 0.01; *** - P ≤ 0.001 for comparisons with T89 by Dunnett’s test; brackets show significant effects of drought for each genotype (t-test, P ≤ 0.05); P values above horizontal lines correspond to contrast analysis. ND, not determined.

In all genotypes, drought significantly reduced stem diameters and these reductions were much smaller in GH10.a and all GH11 lines than in other lines (**Figure 10A**). The impact of drought on wood production and properties in xylan-modified aspen lines was assessed at the stem base by SilviScan. Drought decreased wood diameters in majority of genotypes but not in the two most highly expressing GH11 lines, and all GH11 lines showed reduced drought effects on wood production relative to WW condition compared to T89 (**Figure 10B**). While wood density was increased by drought in T89, either no change or a decrease was observed in xylan-affected lines (**Figure 10C**). Cellulose microfibril angle was increased by drought in the T89 and GT43.a plants but it remained unchanged in xylanase-expressing lines. Remarkably, all GH11-expressing lines had substantially increased microfibril angles in both drought and WW conditions compared to T89 plants. Overall, the wood production and wood properties were less affected by drought in GH11 lines than in the T89.

## Discussion

### Different xylan-impaired *Arabidopsis* genotypes showed common changes in their responses to drought

Characterization of the drought reaction in xylan-altered plants has revealed a plethora of modified reactions compared to wild-type, shedding light on complex plant mechanisms to cope with drought. Remarkably, disregarding line GH11.2 which had minor cell wall composition changes due to low transgene expression, all xylan-modified genotypes exhibited certain developmental alterations that expectedly altered their drought resilience. For example, all lines had increased stomatal density (**Figure 8B**) which could potentially influence transpiration rates. Also, all lines showed better survival rates after the re-watering (**Figure 7D**), including the genotypes without any growth penalty (*irx10*, GH10). Compared to wild-type, all xylan-impaired lines showed reduced rosette growth inhibition by MD but increased inhibition by SD (**Figure 4**), which correlated with increased compared to wild-type SD-induced increase in senescence index PSRI relative to WW condition (**Figure 5C** **;** P_Contrast all_ *_vs_*_Col-0_<0.0001). Overall, xylan impairment leads to better growth resilience under moderate or short-duration stress, but inferior growth maintenance under severe stress. Such a strategy could ultimately lead to a better survival.

The common reaction to drought observed in xylan-impaired *Arabidopsis* is connected to their altered secondary wall, with matrix polysaccharide composition depleted in xylose and enriched in pectin-related sugars (**Tables 1**, **2**). These alterations are in one way or another leading to a weaker secondary wall as supported by the presence of collapsed vessels (the *irregular xylem* phenotype) (**Figure 2C**) (Turner and Somerville, 1997). This kind of defect has been correlated with drought and freezing tolerance (Xin and Browse, 1998; Xin et al., 2007; Bouchabke-Coussa et al., 2008; Lefebvre et al., 2011; Ramírez and Pauly, 2019). The xylanase-expressing genotypes characterized here exhibited not only the *irx* phenotype in common with secondary wall mutants but also better drought survival. These phenotypes of secondary wall mutants were demonstrated to be at least partially dependent on the biosynthesis of carlactone – an intermediate compound in the strigolactone biosynthetic pathway (Ramírez and Pauly, 2019), which was shown to mediate these responses downstream the cell wall biosynthesis defect. These observations support the conclusion that secondary wall defects are mediated through specialized signaling pathway (Hernández-Blanco et al., 2007; Ratke et al., 2018) leading to altered cellular and physiological responses.

### Drought responses of *irx14* and *irx10* mutants correlated with their lignin content

We showed that the better survival rate after re-watering following uncontrolled drought observed in *irx14* (**Figure 7**), and previously reported by Keppler and Showalter (2010), was not caused by reduced growth and thus reduced water usage (Juenger and Verslues, 2023) since the soil water content in pots with *irx14* plants decreased during drought at the same pace as for Col-0. Moreover, we found that *irx14* has increased ability to cope with moderate and severe drought compared with other xylan-impaired lines as supported by many different parameters. Controlled drought exposures indeed showed that among all genotypes studied here, only *irx14* maintained rosette growth under MD, slowed down growth and reduced water content less than wild-type under SD (**Figures 4**, **5B**) and responded to MD by changing F_v_/F_m_ earlier than the wild-type (**Figure 5A**; **Supplementary Figure S3A**). This indicates that the photosystem II electron transport chain adapts to drought stress in this mutant in advance (Woo et al., 2008; Guo and Tan, 2015), enabling it to react faster thus cope with the stress better. Similar trend in F_v_/F_m_ ratio was observed in *irx10* mutant, but it could cope better with drought than wild-type only when the stress was moderate. The similarity in responses of the two mutants to MD is shown by their clustering together in MD but not in SD (**Figure 6**), when only *irx14* showed better relative growth performance compared to wild-type. Two other specific features of *irx14* not observed in *irx10* or other xylan-impaired genotypes were: the normal xylem conductivity to the side shoots (whereas it was defective in other xylan-impaired genotypes) (**Figure 8D**) and the increased xylem lignin content while other genotypes had decreased lignin contents (**Table 2**). Whereas these traits could be connected, it remains elusive why this mutant shows such unique traits not found in other xylan-deficient mutants. It still remains to be investigated if *IRX14* could complement increased lignin content of *irx14* (Brown et al., 2007; Wu et al., 2010; Lee et al., 2012; Ren et al., 2014), which could be responsible for exceptional drought resilience of *irx14* (Bang et al., 2019; Tu et al., 2020).

### Subtle differences in drought responses were observed between xylanase-expressing *Arabidopsis* and *irx* mutants

The apoplast-targeted endo-β-1,4-xylanases induced sometimes specific drought responses, different than those of *irx* mutants. Indeed under MD, the xylanase-expressing lines clustered separately from *irx* mutants (**Figure 6A**). Notably, the PSRI values were always significantly higher in *irx* mutants when contrasted to xylanase lines (**Figure 5C**, supported by P_Contrast GH_ *_vs irx <_* 0.0001 for each WW, MD and SD condition) and increase in PSRI under SD compared to WW conditions tended to be less pronounced in these mutants (P_contrast GH10.1, GH10.2, and GH11.1_ *_vs irx _<*0.002). This indicates that leaves of xylanase-expressing plants have reduced carotenoid:chlorophyll ratio corresponding to lower senescence. Delayed leaf senescence was shown to play a role in drought tolerance (Rivero et al., 2007) and it could constitute a specific mechanism of increased drought resilience in xylanase-expressing lines.

Subtle differences between plants with synthetically- and post-synthetically modified xylan are to be expected. Altered xylan backbone biosynthesis in *irx* mutants could lead to substrate imbalance in the Golgi and the subsequent substrate-driven changes in the products by enzymatic reactions, as it was most likely causing altered xylan acetylation in *gux* biosynthetic mutants and changes in xylan glucuronidation in *tbl* mutants (Chong et al., 2014; Lee et al., 2014). These imbalances are likely to be sensed by plants leading to specific metabolic and physiological adjustments. On the other hand, xylanase-expressing plants could be affected by the transgene product alone, which could be perceived as a PAMP (Enkerli et al., 1999; Zhang et al., 2014), or by the product of xylanase activity – xylobiose – which can be perceived as a DAMP (Dewangan et al., 2023).

### GH10 and GH11 xylanases differentially affected drought responses of *Arabidopsis* and aspen

Whereas both GH10 and GH11 enzymes are endo-β-1,4-xylanases, their specificities slightly differ (Biely et al., 1997, 2015; Kojima et al., 2022). In general, only GH10 xylanases can hydrolyze directly β-1,4-glycosidic linkage in the xylan backbone adjacent to GlcA substituent at the nonreducing end, whereas GH11 xylanases display higher activity. This results in a different oligosaccharide spectrum that the two xylanases release from the secondary wall xylan (Persson et al., 2007), which could have different DAMP-inducing potency (Dewangan et al., 2023).

In *Arabidopsis*, the effect of GH11 (considering line GH11.1) on cell wall composition was more pronounced and different than the effect of GH10 (**Tables 1**, **2**). Also, GH11 had more detrimental effect on rosette area in MD (**Figure 4A**, P_Contrast GH10_ *_vs_*_GH11.1_< 0.0004) and early rosette growth rate (expressed in % of WW) in MD (**Figure 4B** P_Contrast GH10_ *_vs_*_GH11.1_< 0.09). This was correlated with both a lower F_v_/F_m_ ratio in MD (**Figure 5A**, P_Contrast GH10_ *_vs_*_GH11.1_< 0.05), a higher water loss index in MD (**Figure 5B**, P_Contrast GH10_ *_vs_*_GH11.1_< 0.003), and a higher senescence index in all conditions (**Figure 5C**, P_Contrast GH10_ *_vs_*_GH11.1_< 0.0003). In addition, it was supported by severely reduced stem conductivity (**Figures 8E, F**) and transpiration during drought (**Figure 7B**, P_Contrast GH10_ *_vs_*_GH11.1_<0.02) in GH11- compared to GH10-expressing plants. Moreover, line GH11.1 exhibited much larger pore size than that of GH10-expressing lines regardless the ABA treatment (**Figure 8C**, P_Contrast GH10_ *_vs_*_GH11.1_<0.0001 for all treatments). The smaller stomata could help to mitigate the effect of the drought by increasing WUE and leaf water retention (Xu and Zhou, 2008; Zhao et al., 2015) which could have perhaps mediated a better performance of GH10 lines compared to GH11.

Opposite effects of the two xylanases on drought resilience have been observed in aspen, where GH11-expressing plants appeared more drought tolerant compared GH10-expressing ones. This was particularly striking when we analyzed the change in the rate of stem elongation (**Figure 9C**), xylem production (**Figure 10B**) and xylem density (**Figure 10C**) under drought. Likewise, the abnormally high leaf pseudo-temperature registered for GH10.b in WW conditions and no change in the drought conditions (**Figure 9F**) indicate low transpiration rates and impaired reaction to drought in this line. The latter is supported by a reduced effect of ABA on stomatal closure in *Arabidopsis* line GH10.1, compared to GH11 or Col-0, so with this regard, effects of GH10 might share similarities between the two species. Opposite effects of the two xylanases on plant growth in aspen and *Arabidopsis* could be related to different parameters that were measured, the rosette area in *Arabidopsis* and the stem height and diameter growth in aspen. It is also possible that the differences are caused by severity of stress signaling. Aspen lines expressing xylanases exhibited growth penalty under WW conditions, that was not seen in *Arabidopsis*. The aggravation of the impact of xylanases in aspen was expected as the altered xylan structures would affect proportionally more cells of its body compared to herbaceous plant, resulting in a stronger stress signaling. Finally, there are probably different mechanisms for coping with severe drought stress in plants with different lifestyles, particularly when considering annual herbaceous and perennial woody plants.

## Conclusions and perspectives

Our research has shown that xylan integrity in the secondary cell walls is an important factor contributing to plant development under normal and drought stress conditions. By comparing different plant models transformed with the same fungal xylanases and targeting them to secondary walls or affected during similar stage of xylan backbone biosynthesis, we identified similar effects of different xylan impairment within a species as well as differences among different types of impairment within a species, and differences between woody and herbaceous species. Molecular mechanisms mediating drought resilience by these specific mechanisms are currently unknown and deserve investigations. We have shown that while obtaining transgenic crops with improved performance in normal and drought condition is feasible (e.g. GT43BC lines), increased drought resistance seems to be correlated with growth penalty under well-watered conditions and breaking this correlation is still a task ahead. Fine-tuning plants to survive a range of conditions is a challenge that needs to be addressed with very thorough attention as we demonstrate the plant’s reaction to subtle changes in the xylan could lead to various physiological adjustments. This work also illustrates that stress resilience might be connected to secondary cell wall integrity signaling which needs to be addressed when generating crops that effectively fulfill the specified objectives. Understanding this mechanism acting during secondary cell wall formation would be a breakthrough in the field of wood biology (Zhao et al., 2013; Ratke et al., 2018; Gallego-Giraldo et al., 2020; Dewangan et al., 2023). Moreover, elucidation of molecular pathways of these responses should be addressed in evolutionary context as even closely-related species can adopt different stress managing mechanisms (Marín-de la Rosa et al., 2019).

## Data availability statement

The original contributions presented in the study are included in the article/**Supplementary Material**. Further inquiries can be directed to the corresponding author.

## Author contributions

FRB: Conceptualization, Formal analysis, Investigation, Methodology, Visualization, Writing – original draft. EC: Formal analysis, Investigation, Methodology, Supervision, Writing – review & editing. END: Data curation, Formal analysis, Software, Visualization, Writing – review & editing. IG: Methodology, Writing – review & editing. JU: Investigation, Writing – review & editing. GP: Investigation, Writing – review & editing. HD: Investigation, Writing – review & editing. DJ: Investigation, Writing – review & editing. ZY: Investigation, Methodology, Writing – review & editing. MD-M: Investigation, Methodology, Writing – review & editing. ERM: Conceptualization, Methodology, Writing – review & editing. GS: Methodology, Writing – review & editing. LG: Investigation, Methodology, Supervision, Writing – review & editing. EJM: Conceptualization, Funding acquisition, Project administration, Supervision, Writing – review & editing.
