# Supplementary material for: Integrity of xylan backbone affects plant responses to drought
Source: Front Plant Sci. 2024 Jun 25;15:1422701. doi: 10.3389/fpls.2024.1422701 (PMC11231379; doi:10.3389/fpls.2024.1422701)
Supplement: Supplementary file 1 [file DataSheet_1.docx]

Supplementary Material

Integrity of xylan backbone affects plant responses to drought

Félix R. Barbut, Emilie Cavel, Evgeniy N. Donev, Ioana Gaboreanu, János Urbancsok, Garima Pandey, Hervé Demailly, Dianyi Jiao, Zakiya Yassin, Marta Derba-Maceluch, Emma R. Master, Gerhard Scheepers, Laurent Gutierrez, Ewa J. Mellerowicz^*^

*** Correspondence:** Ewa Mellerowicz: [Ewa.Mellerowicz@slu.se](mailto:Ewa.Mellerowicz@slu.se)

## Supplementary Figures:

**Supplementary Figure S1. Recombinant GH10:eGFP and GH11:eGFP proteins were targeted to cell walls.**

**Supplementary Figure S2. Xylan impairment did not affect the anatomy of eight-week-old hypocotyls in *Arabidopsis* except for the *irx9* mutant**.

**Supplementary Figure S3. Kinetics of fluorescence and reflected light spectral parameters revealed subtle differences in drought responses of xylan-modified *Arabidopsis* lines and the wild-type.**

**Supplementary Figure S4.** **Xylan-modified *Arabidopsis* lines exhibited altered changes in greenness in response to drought.**

**Supplementary Figure S5. Water usage of xylan-modified aspen lines under different watering conditions reflects their growth.**

**
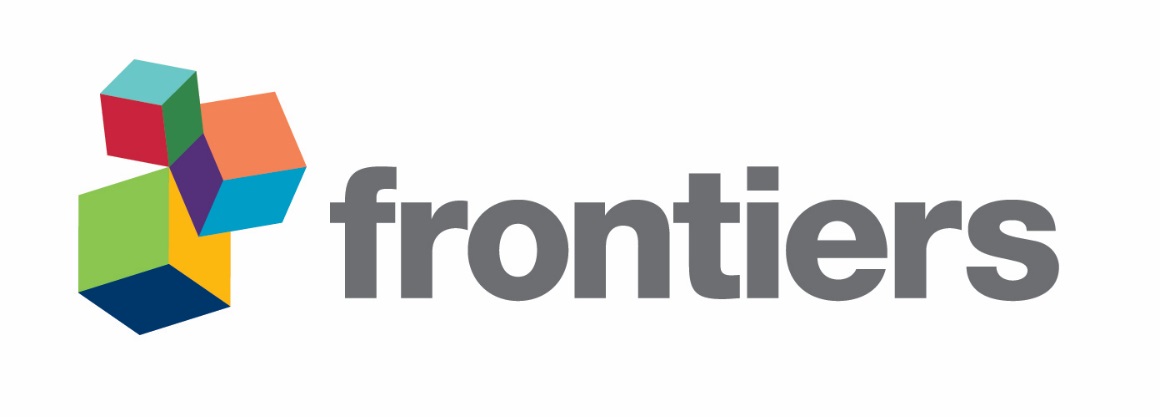
**

**Supplementary Figure S1.** **Recombinant GH10:eGFP and GH11:eGFP proteins were targeted to cell walls.** (**A**) Schematics of constructs employed. (**B**) Lambda scans (right figure) of the signal detected in GFP channel in selected regions within cell wall of plasmolyzed cells (left figure, arrows) showing expected intensity distribution for eGFP. No fluorescence signal was detected in wild-type plants used as negative control (**C**). Scale bar 10 µm. SP: Signal peptide; eGFP: enhanced green fluorescent protein.

**Supplementary Figure S2.** **Xylan impairment did not affect the anatomy of eight-week-old hypocotyls in *Arabidopsis* except for the *irx9* mutant**. **(A)** Phloroglucinol stained hypocotyl cross-sections. x1=xylem 1, x2=xylem 2, bar = 500 µm. **(B)** Xylem 2 area relative to total secondary xylem. Means ± se, n=4. * - P≤0.05 for comparisons with Col-0 by Dunnett’s test.


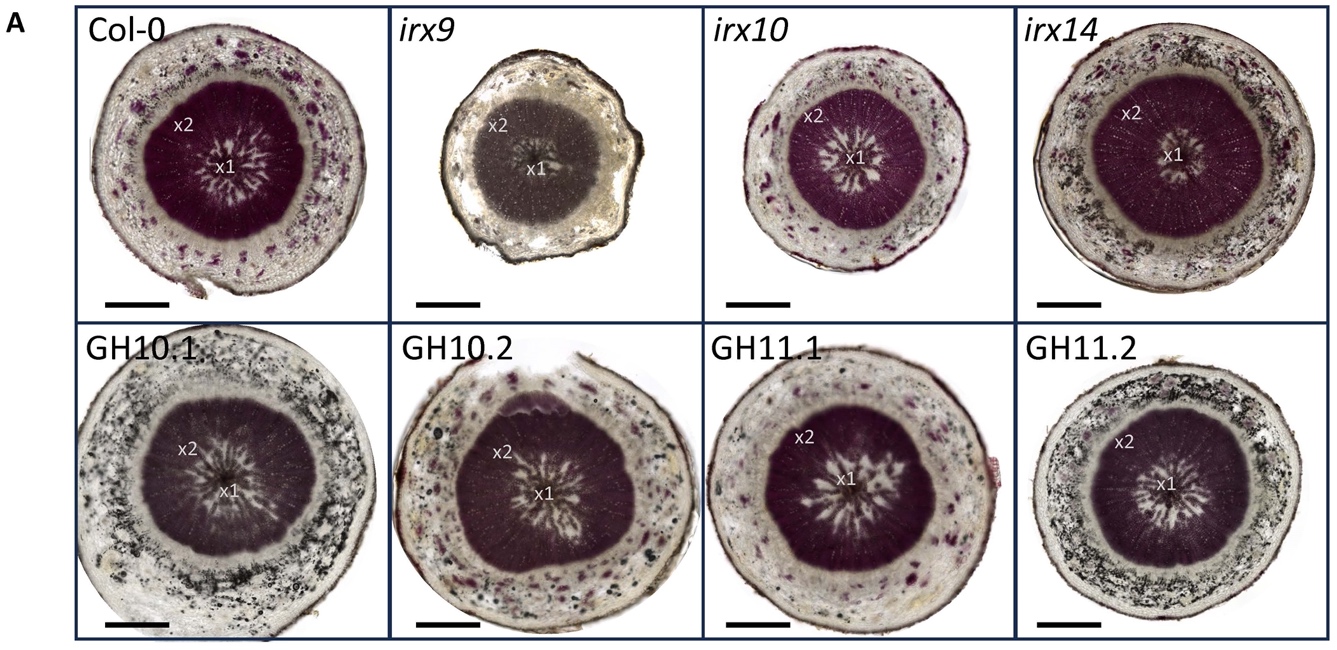

**Supplementary Figure S3.** **Kinetics of fluorescence and reflected light spectral parameters revealed subtle differences in drought responses of xylan-modified *Arabidopsis* lines and the wild-type.** Kinetics of F_v_/F_m_ ratios (**A**), Water Loss Index (WLI) divided by Normalized Difference Vegetation Index (NDVI) (**B**), Plant Senescence Reflectance Index (PSRI) (**C**)**,** and Photochemical Reflectance Index (PRI) (**D**) from 21^st^ to 38^th^ day after sowing under well-watered (WW), moderate drought (MD) and severe drought (SD) conditions. Data are means ± se; n: 24-37.

Fig. S3 continues on the next page.

Fig. S3 (cont.).

**Supplementary Figure S4.** **Xylan-modified *Arabidopsis* lines exhibited altered changes in greenness in response to drought.** (**A**) Kinetics of proportions among different greenness hues. Color segmentation of images from RGB camera discerned nine hues described by their RGB coordinates shown below the area graphs. (**B**-**C**) The proportion of hue 3+5 (**B**) and hue 4+6 (**C**) at different watering conditions (upper row) and in MD or SD conditions relative to WW conditions. WW – well-watered; MD – moderate drought; SD – severe drought. Data in (**B**-**C**) are means ± se; n: 24-37; ・- P≤0.1; * - P≤0.05; ** - P≤0.01; *** - P≤0.001 for comparisons with Col-0 by Dunnett’s test; different letters indicate significant treatment effect within the same genotype (Tukey’s test, P≤0.05).

**Supplementary Figure 5.** **Water usage of xylan-modified aspen lines under different watering conditions reflects their growth.** Mean daily water usage per plant is based on four-day means during drought treatment (D) and in well-watered (WW) conditions from 17 to 61 days after planting (upper row) and in D relative to WW conditions (lower row). Means ± se; n= 6-11; * - P≤0.05; ** - P≤0.01; *** - P≤0.001 for comparisons of end values of transgenic lines with T89 by Dunnett’s test. Arrows indicate the day when the target drought level was reached.
